# Neutral iodotriazoles as scaffolds for stable halogen-bonded assemblies in solution†
†Electronic supplementary information (ESI) available: Details of computational methods, synthesis and characterisation data for all compounds are provided. CCDC 1442418–1442421. For ESI and crystallographic data in CIF or other electronic format see DOI: 10.1039/c6sc01974a



**DOI:** 10.1039/c6sc01974a

**Published:** 2016-06-23

**Authors:** Leonardo Maugeri, Julia Asencio-Hernández, Tomáš Lébl, David B. Cordes, Alexandra M. Z. Slawin, Marc-André Delsuc, Douglas Philp

**Affiliations:** a School of Chemistry and EaStCHEM , University of St Andrews , North Haugh St Andrews , Fife KY16 9ST , UK . Email: d.philp@st-andrews.ac.uk ; Fax: +44 (0)1334 463808 ; Tel: +44 (0)1334 467264; b Institut de Génétique et de Biologie Moléculaire et Cellulaire , INSERM U596 , CNRS UMR 7104 , Université de Strasbourg , 1 rue Laurent Fries , 67404 Illkirch-Graffenstaden , France

## Abstract

The halogen bond (XB) donor properties of neutral 1,4-diaryl-5-iodo-1,2,3-triazoles are explored using a combination of computational and experimental results and are shown to be competitive in halogen bonding efficiency with the classic pentafluoroiodobenzene XB donor. The S_N_Ar reactivity of these donors permits the facile assembly of an iodotriazole functionalised with a 3-oxypyridine XB acceptor, thus generating a molecular scaffold capable of undergoing dimerisation through the formation of two halogen bonds. The formation of this halogen-bonded dimer is demonstrated by ^1^H and DOSY NMR experiments and a plausible structure generated using DFT calculations.

## Abstract 2

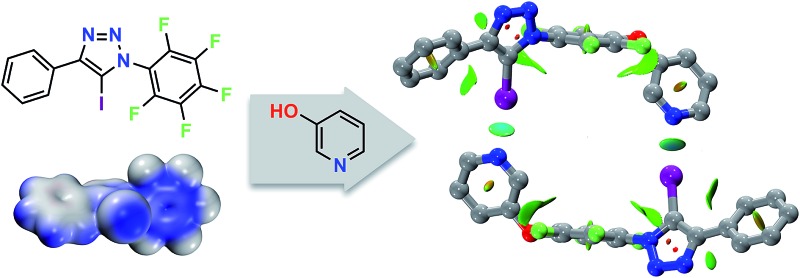
Computational and experimental data are used to demonstrate that the halogen bond (XB) donor properties of neutral 1,4-diaryl-5-iodo-1,2,3-triazoles are competitive with the classic pentafluoroiodobenzene XB donor.

## Introduction

The non-covalent interaction between an electron deficient halogen atom and an electron donor has found^1^ a clear definition in the term halogen bonding. Halogen bonds (XBs) have proven to be a powerful tool in a wide range of chemistries. The contribution of Metrangolo, Resnati and co-workers has revolutionised^2^ the conception of XB, making it a valuable tool in all the areas of chemistry where molecular recognition plays a central role.^3^ A significant effort has also been invested in unravelling the fundamental structural and energetic features of XBs. Computational^4^ and experimental^5^ studies made significant contribution to the understanding of the so-called “σ-hole”^4*b*^ – a zone of low electron density displaying a positive electrostatic potential. Simplifying XBs to merely electrostatic interactions is, however, reductive: the factors contributing to the stability of a XB are a convolution of electrostatic,^4*a*^ polarisation,^6^ charge transfer^7^ and dispersion^8^ forces and the role played by each of these factors is influenced significantly by the molecular components involved and the medium where the interaction takes place. It is generally recognised though that the ability of a halogen atom to participate in a XB increases with the electron withdrawing effect of the group to which the halogen is attached. The plethora of halogenated molecules constitutes a huge set of XB donors. Among these, a simple demarcation in XB ability can be made between cationic and neutral XB donors. Cationic XB donors are generally halogenated (mainly Br and I) five or six membered nitrogen heterocycles, where one of the nitrogen atoms is quaternarised with an alkyl group. The interaction of such XB donors with XB acceptors is in fact defined^9^ as ‘charge-assisted XB’ and these interactions are significantly stronger than those between two neutral XB partners. Beer and co-workers, for example, have designed and synthesised^10^ a variety of macrocycles, catenanes and rotaxanes incorporating halotriazolium and haloimidazolium units able to perform anion sensing in organic and aqueous media *via* charge-assisted XB. Pyridinium^11^ and imidazolium^12,13^ XB donors have been used extensively by the group of Huber to catalyse C–Br bond cleavage reactions in organic media.

On the other hand, when a polarisable halogen atom is bonded to a neutral organic backbone, its ability to act as a XB donor depends greatly on the electronic properties of the organic residue. It is, therefore, not surprising that the most common neutral XB donors are perfluorohalocarbons (PFHCs). Whether aromatic or aliphatic, these compounds are now considered ‘iconic’ XB donors. In 2002, Metrangolo, Resnati *et al.* reported^14^ the first semiquantitative evaluation of XB in solution using 1,2-dibromo and 1,2-diiodo tetrafluoroethanes as the XB donors. Taylor *et al.* have successfully measured^15^ the strength of XBs formed by a set of variously substituted iodoperfluorobenzenes XC_6_F_4_I in solution using ^19^F NMR spectroscopy. The association strength could be correlated to the *σ* Hammett parameter for the X substituent. More recently multivalent PFHC-based XB donors have been successfully exploited in molecular recognition^16^ and catalysis.^17^

The quest for new and less conventional organic XB donors has been tackled^18^ by several research groups and haloalkenes,^19^ haloalkynes,^20^ *N*-haloimides^21^ and halogenated metallic complexes^22^ have been found to be strong XB donors in the solid state. Iodoalkynes have been shown^23^ to behave as XB donors in solution as well as in the solid state.

In this paper, we describe the development of an underexplored^24^ class of iodinated scaffolds, 1,4-diaryl-5-iodo-1,2,3-triazoles, as a progenitor for assemblies supported by XBs. The XB properties have been examined computationally, in the solid state and in solution. During the course of these studies, a serendipitous discovery led us to design a system containing both a diaryliodotriazole and a Lewis base, thus generating a molecule capable of undergoing dimerisation through self-complementary XBs. A detailed NMR study of the self-assembled dimer in solution has allowed us to evaluate the efficiency of chelate cooperativity on the dimerisation process.

## Results and discussion

**Fig. 1:**
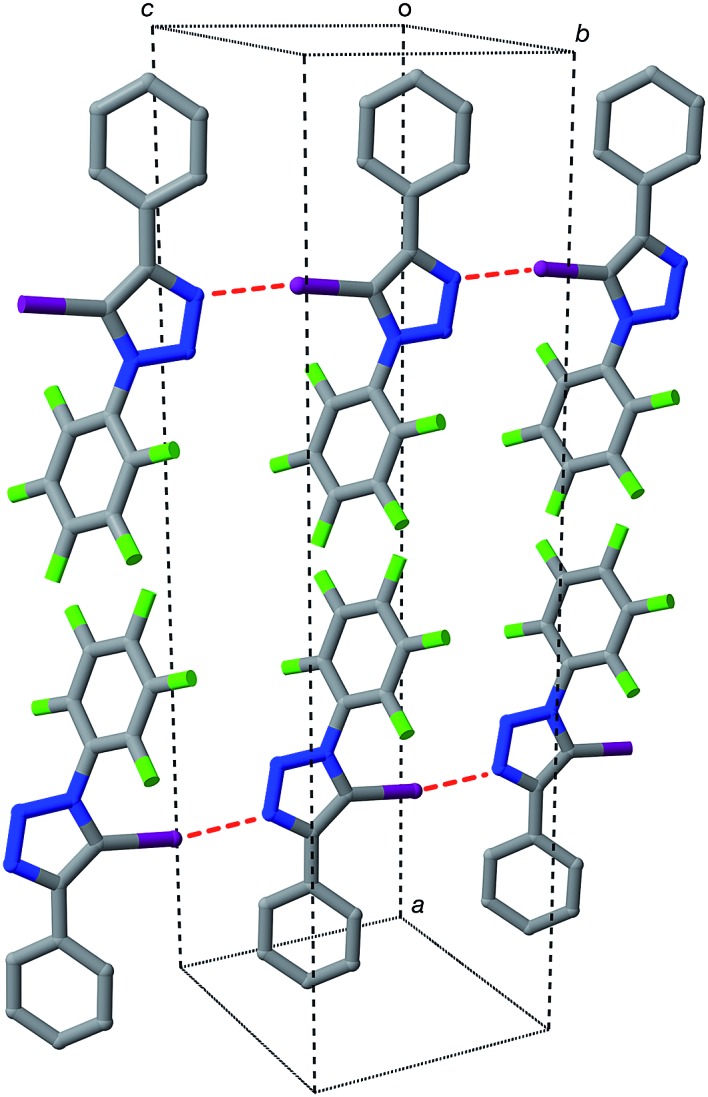
Solid state structure of diaryl-5-iodo-1,2,3-triazole **1** determined from single crystal X-ray diffraction data. Potential halogen bonds are marked in red (*r*(N···I) = 2.973 Å; ∠C–I···N = 168.9°). Atom colouring: C atoms = grey, N atoms = blue, F atoms = light green, I atoms = purple. Hydrogen atoms are omitted for clarity.

Iodotriazole **1** can be prepared readily and in high yield through the Cu-catalysed reaction of pentafluorophenyl azide and iodophenylacetylene. The solid state structure (Fig. 1) of compound **1**, determined by single crystal X-ray diffraction, exhibits antiparallel tapes in which the molecules are connected by a series of short, nitrogen to iodine contacts (*r*(N···I) = 2.973 Å) indicative of the presence of a halogen bond between the iodotriazole rings.

The presence of these close contacts in the solid state structure of **1** suggested that appropriately designed diaryl-5-iodo-1,2,3-triazoles could function as viable XB donors.

**Fig. 2:**
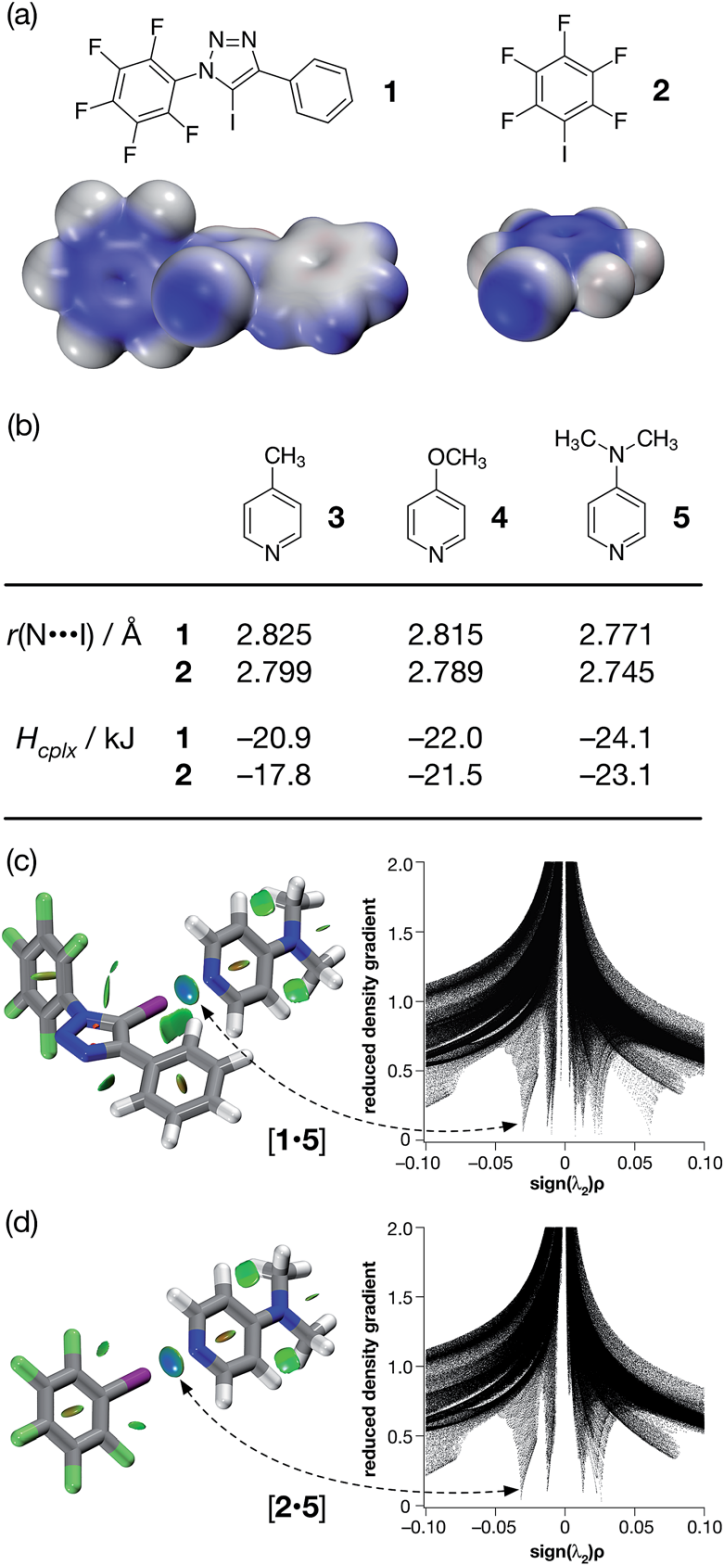
(a) Calculated electrostatic potential surfaces for compounds **1** and **2** (TPSSh/def2-TZVP). Colour scale: positive (blue = +0.08) → neutral (white) → negative (red = –0.08). (b) Calculated pyridine ring N···I distances and interaction energies (*H*_cplx_, TPSSh/def2-TZVP enthalpies of complexation at 298 K, see ESI† for details) for a series of complexes between halogen bond donors **1** and **2** and pyridine-based acceptors. Visualisation of the interaction between 4-(dimethyl amino)pyridine **5** and (c) compound **1** and (d) compound **2**. Left: intermolecular interaction isosurfaces generated by NCIPLOT^26^ for *s* = 0.5 and –0.05 < sign(*λ*_2_)*ρ* < 0.05 (colour scale: attractive (blue) → repulsive (red)). Right: plots of sign(*λ*_2_)*ρ vs.* reduced density gradient highlighting the favourable interaction corresponding to the halogen bond at sign(*λ*_2_)*ρ* ∼ –0.035. Atom colouring: C atoms = grey, N atoms = blue, O atoms = red, F atoms = light green, I atoms = purple.

In order to gain some insight into the potential interactions between this class of compounds and XB acceptors, we performed a series of calculations that compared the interaction of iodotriazole **1** and pentafluoroiodobenzene **2** with a series of pyridine-based XB acceptors (Fig. 2). These calculations were performed at the TPSSh/def2-TZVP level of theory.

Examination of the electrostatic potential of **1** (Fig. 2a) shows that this compound possesses an area of significant positive potential associated with the iodine atom. This area of positive potential is similar in magnitude to that calculated for pentafluoroiodobenzene **2** – the iconic^25^ XB donor. These results suggest that iodotriazole **1** should interact strongly with suitable electron donors. Indeed, the calculated geometries for the complexes formed between the three pyridines shown in Fig. 2b and compound **1** all possess N···I distances much shorter than the sum of the van der Waals radii and significantly negative enthalpies of complexation at 298 K (Fig. 2b). These structural and energetic parameters are all similar to those for the complexes formed between the well-known halogen bond donor **2** and the same set of pyridines. Natural bond order (NBO) analyses (see ESI† for details) demonstrated that, in all six complexes, there are significant interactions between the nitrogen lone pair and the σ* orbital associated with the C–I bond present in the donor. Intermolecular interaction isosurfaces, generated^26^ through an analysis of the reduced gradient of the electron density, have been used^27^ extensively to identify non-covalent interactions that stabilise intermolecular complexes. Analyses of the complexes [**1·5**] (Fig. 2c) and [**2·5**] (Fig. 2d) using this method (see ESI† for details) reveal significant low density, low gradient regions that are consistent with the presence of a halogen bond between the interacting partners in both complexes. The similarities between the results for [**1·5**] and [**2·5**] suggest that the neutral iodotriazole might be used interchangeably with the perfluorinated iodobenzene as a halogen bond donor.

Encouraged by these computational results, we examined the interaction of iodotriazole **1** and the pyridine-based XB acceptors **3** and **5** in solution. Initially, we examined the interaction of **1** and 4-methylpyridine **3** in *d*_8_-toluene. Titration of increasing amounts of **3** into a solution of **1** in *d*_8_-toluene did not result in any significant chemical shift changes in the ^19^F NMR spectrum of **1**. However, when the titration was performed again, this time titrating increasing amounts of **1** into a solution of **3** in *d*_8_-toluene at 293 K, a series of ^1^H–^15^N HMBC experiments revealed significant upfield ^15^N chemical shift changes for the pyridine ring nitrogen atom. The ^15^N chemical shift data were fitted^28^ to a 1 : 1 binding model for the complex [**1·3**] affording (see ESI† for details) a stability constant for this complex in *d*_8_-toluene at room temperature of 1.67 ± 0.55 M^–1^. For comparison purposes, we repeated this analysis, this time using pentafluoroiodobenzene **2** as the halogen bond donor. The ^15^N chemical shift data from this experiment were once again fitted^28^ to a 1 : 1 binding model for the complex [**2·3**] (see ESI† for details) affording a stability constant for this complex in *d*_8_-toluene at 293 K of 2.67 ± 0.69 M^–1^. These results confirm experimentally the outcome of our calculations – iodotriazole **1** and pentafluoroiodobenzene **2** have similar halogen bond donor abilities towards pyridine acceptors.

**Fig. 3:**
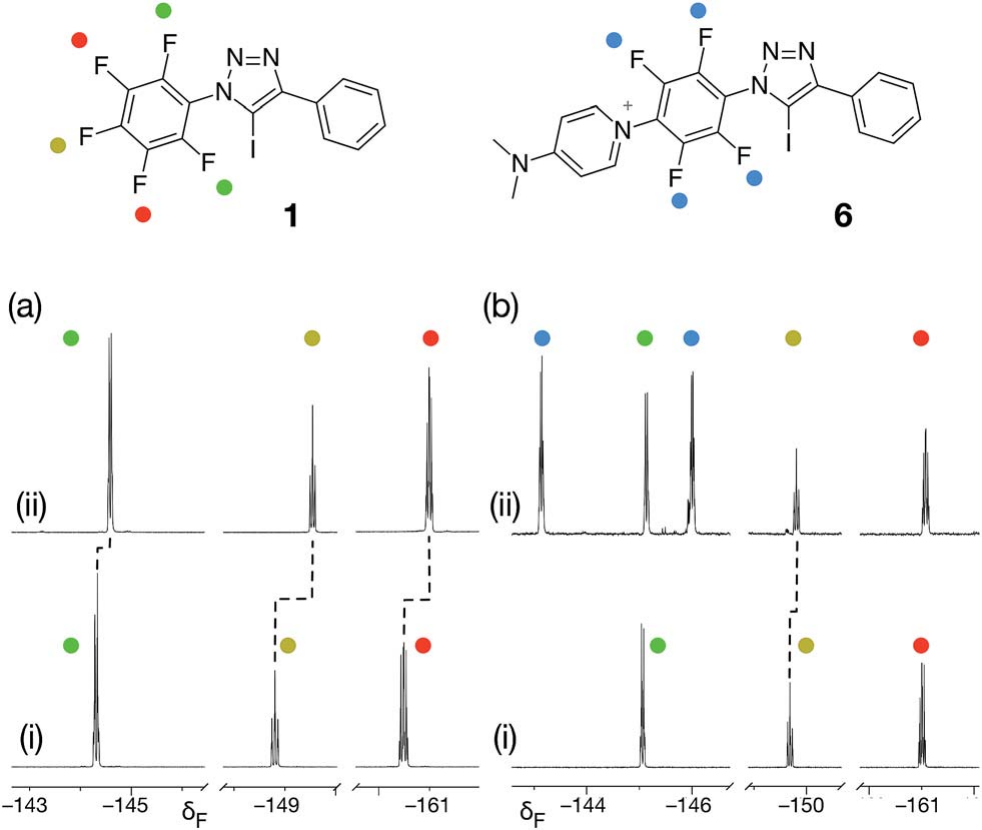
(a) Partial 376.4 MHz ^19^F NMR spectra of (i) a solution of **1** in *d*_8_-toluene and (ii) the same solution after the addition of 10 equivalents of DMAP **5**. (b) Partial 376.4 MHz ^19^F NMR spectra of (i) a solution of **1** in CD_3_CN and (ii) the same solution after the addition of 10 equivalents of DMAP **5**. In both case, dashed lines indicate resonances that show chemical shift changes between (i) and (ii).

From the set of pyridine XB acceptors studied computationally, DMAP **5** was predicted to form the most stable complexes. When we performed an experiment where increasing amounts of **5** were titrated into a solution of **1** in *d*_8_-toluene, significant chemical shift changes in the 376.4 MHz ^19^F NMR spectrum of **1** were observed (Fig. 3a). In particular, the resonance for the fluorine atom *para* to the iodotriazole ring exhibited a significant upfield shift (Fig. 3a, dotted line) from *δ* –148.7 to *δ* –149.6, consistent with the formation of the [**1·5**] complex. However, this chemical shift data could not be fitted to a 1 : 1 binding model for the [**1·5**] complex and close examination of the NMR samples revealed that a precipitate had formed in all samples. Clearly, the formation of the [**1·5**] complex was accompanied by a concomitant chemical transformation.

Intrigued by the presence of this precipitate, we repeated this titration experiment using CD_3_CN as the solvent. In this case, no precipitate was formed. However, the 376.4 MHz ^19^F NMR spectrum revealed a new set of resonances at *δ* –143.1 and *δ* –145.9 (Fig. 3b, blue circles) that were consistent with the presence of a 1,4-disubstituted tetrafluorobenzene ring. We attributed these resonances to the presence of the insoluble pyridinium salt **6**. This product arises from the nucleophilic aromatic substitution^29,30^ (S_N_Ar) of the fluorine *para* to the triazole ring in **1** by DMAP **5** and analysis of the precipitate formed in *d*_8_-toluene confirmed its identity as the pyridinium salt **6**.

**Fig. 4:**
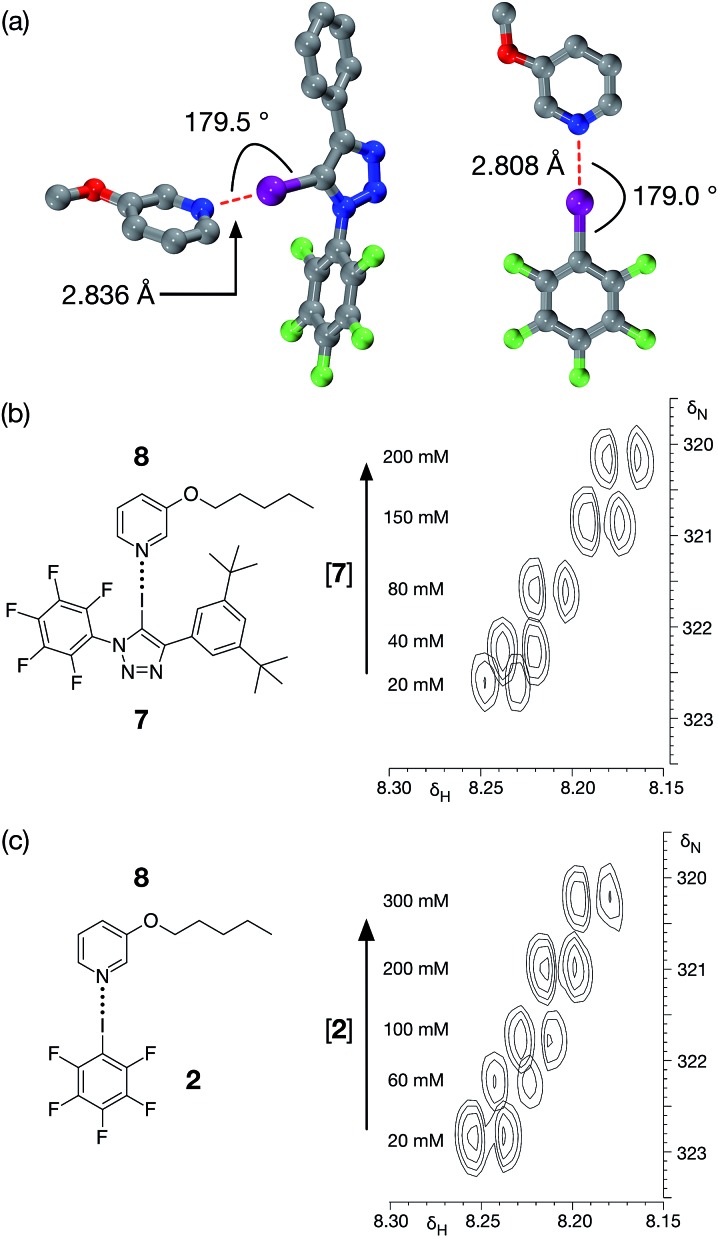
(a) Calculated structure (TPSSh/def2-TZVP) for the complexes formed between **1** and 3-methoxypyridine (left) and between **2** and 3-methoxypyridine (right). Halogen bonds are marked in red. For the complex of 3-methoxypyridine with **1**: (*r*(N···I) = 2.836 Å; ∠C–I···N = 180.0°). For the complex of 3-methoxypyridine with **2**: (*r*(N···I) = 2.808 Å; ∠C–I···N = 179.0°). Carbon atoms are coloured grey, nitrogen atoms are coloured blue, fluorine atoms are coloured green and iodine atoms are coloured purple. Hydrogen atoms are not shown. (b) Partial 700.1 MHz ^1^H–^15^N HMBC correlations of a 20 mM solution of pyridine **8** in *d*_8_-toluene with increasing amounts (0 to 200 mM) of iodotriazole **7** added to the solution. (c) Partial 700.1 MHz ^1^H–^15^N HMBC correlations of a 20 mM solution of pyridine **8** in *d*_8_-toluene with increasing amounts (0 to 200 mM) of pentafluoroiodobenzene **2** added to the solution.

Although DMAP **5** is potentially the best halogen bond acceptor, it is clearly much too reactive towards the perfluorinated aromatic ring present in **1**. We therefore turned to alkoxypyridines as halogen bond acceptors. We performed a series of calculations examining the interaction of iodotriazole **1** with 3-methoxypyridine at the TPSSh/def2-TZVP level of theory. These calculations reveal (Fig. 4a, left) a complex that is very similar in structure (*r*(N···I) = 2.836 Å, ∠C–I···N = 179.5°) and with a similar calculated enthalpy of complexation at 298 K (–23.6 kJ mol^–1^) to that formed between **1** and 4-methylpyridine **3**. Titration of **7**, a more soluble variant of **1**, into a solution of 3-pentyloxypyridine **8** in *d*_8_-toluene at 298 K resulted in small, but significant, chemical shift changes in the 700.1 MHz ^1^H NMR spectrum for the resonances arising from the pyridine ring protons of **8**. A series of ^1^H–^15^N HMBC experiments (Fig. 4b), performed on the same sample set, also reveal significant ^15^N chemical shift changes for the pyridine ring nitrogen atom. Both the ^1^H and the ^15^N chemical shift data were fitted^28^ to a 1 : 1 binding model for the complex [**7·8**] affording (see ESI† for details) a stability constant for this complex in *d*_8_-toluene at 293 K of 1.44 ± 0.24 M^–1^.

For comparison purposes, we also evaluated the stability of the complex formed between pentafluoroiodobenzene **2** and 3-methoxypyridine at the same level of theory. These calculations reveal (Fig. 4a, right) a complex with a halogen bond that is similar in geometry (*r*(N···I) = 2.808 Å, ∠C–I···N = 179.0°) and a similar calculated enthalpy of complexation at 298 K (–19.6 kJ mol^–1^) to the complex formed between 3-methoxypyridine and iodotriazole **1**. Analysis of a titration of **2** into a solution of 3-pentyloxypyridine **8** in *d*_8_-toluene using a series of 700.1 MHz ^1^H–^15^N HMBC experiments (Fig. 4c) reveal significant ^1^H and ^15^N chemical shift changes in the pyridine ring. These data were fitted^28^ to a 1 : 1 binding model for the complex [**2·8**] affording a stability constant for this complex in *d*_8_-toluene at room temperature of 1.40 ± 0.17 M^–1^ (see ESI† for details).

It is clear from these data that the interactions between both **2** and **8** and **7** and **8** are is not particularly strong in *d*_8_-toluene at 298 K. However, we reasoned that the reactivity observed between **1** and nucleophiles, such as an amino- or hydroxypyridine, should allow us to construct rapidly a molecule that possessed both a pyridine ring and an iodotriazole. Such a molecule would be self-complementary and could, potentially, benefit from cooperative binding^31^ between the self-complementary recognition sites, thus forming a halogen-bonded dimer.

**Fig. 5:**
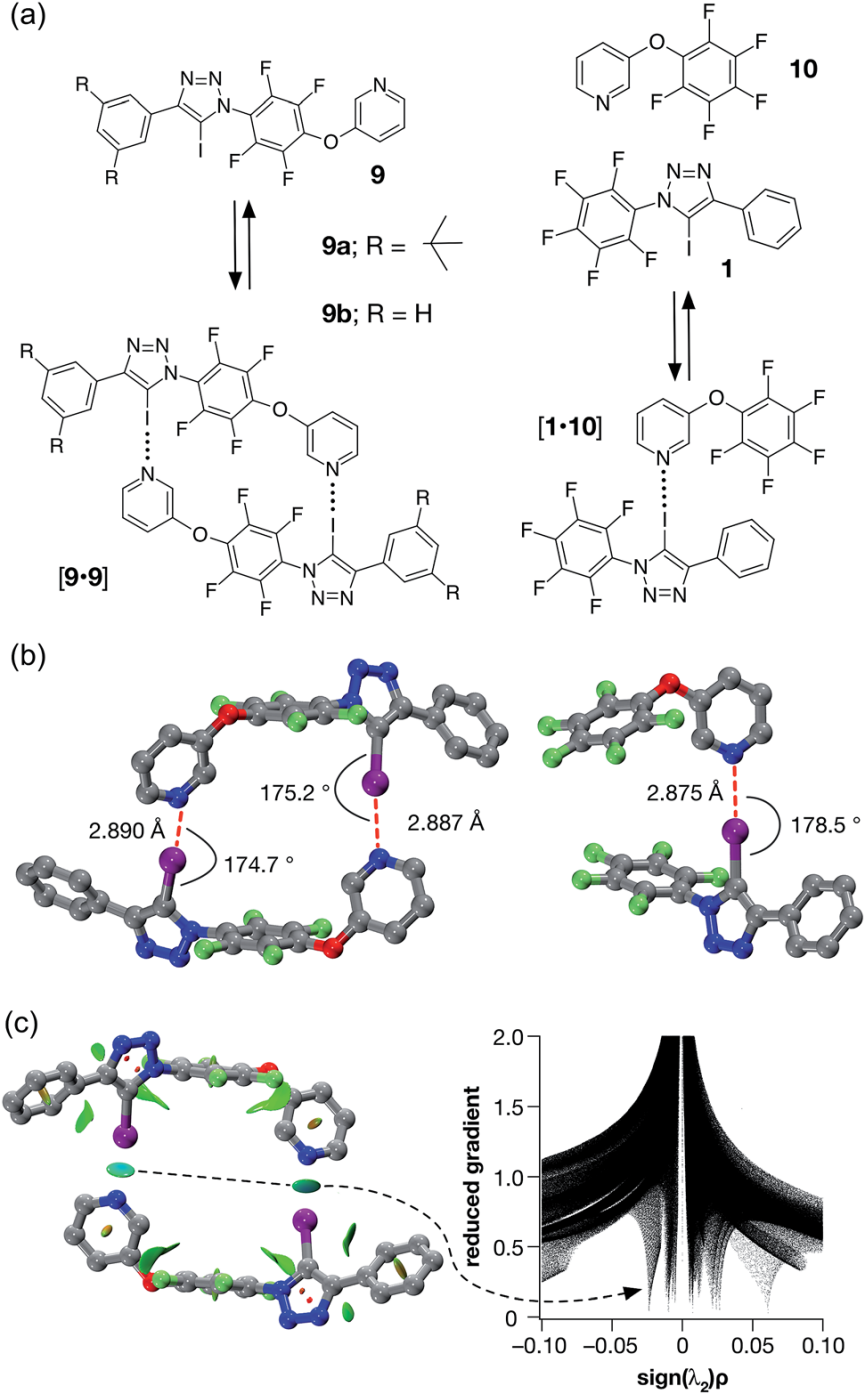
(a) Iodotriazole **9b** provides a computational model for **9a**, which was prepared synthetically. Both iodotriazoles can dimerise through two halogen bonds to form homodimers. Complex [**1·10**] provides a computational model for the halogen bonds found in the dimers. (b) Comparison of the calculated structures (TPSSh/def2-TZVP) of the [**9b·9b**] dimer and the complex [**1·10**]. (c) Left: intermolecular interaction isosurfaces for the [**9b·9b**] dimer, generated by NCIPLOT,^26^ for *s* = 0.5 and –0.05 < sign(*λ*_2_)*ρ* < 0.05 (colour scale: attractive (blue) → repulsive (red)). Right: plot of sign(*λ*_2_)*ρ vs.* reduced gradient highlighting the favourable interaction corresponding to the halogen bonds at sign(*λ*_2_)*ρ* ∼ –0.035. Atom colouring in molecular structures: C atoms = grey, N atoms = blue, O atoms = red, F atoms = light green, I atoms = purple. H atoms are omitted for clarity.

Accordingly, we designed compound **9a** (Fig. 5a), which was prepared in two steps, in high yield, from 5-(2-iodoethynyl)-1,3-bis(*tert*-butyl)benzene and 1-azido-2,3,4,5,6-pentafluoro benzene (see ESI† for details). We envisaged that **9a** might be able to form a halogen-bonded dimer in which the pyridine ring of one molecule interacts with the iodotriazole of a second molecule. This expectation was supported by calculations (Fig. 5b) at the TPSSh/def2-TZVP level of theory on compound **9b** – identical to **9a** save for the replacement of the *tert*-butyl groups by hydrogen atoms in the interests of computational efficiency.

A doubly halogen-bonded homodimeric structure was located that possessed approximate *C*_2_ symmetry and in which the tetrafluoroaromatic rings in the two molecules of **9b** are rotated by around 120° with respect to each other. The two halogen bonds showed almost identical lengths and geometries (*r*(N···I) = 2.887 Å; ∠C–I···N = 175.2° and *r*(N···I) = 2.890 Å; ∠C–I···N = 174.7°). The calculated enthalpy of dimerisation at 298 K for [**9b·9b**] is –30.6 kJ at this level of theory. Comparison of [**9b·9b**] with the corresponding monodentate interaction – as represented by the calculated structure of the complex formed between **1** and pyridine **10** at the same level of theory (Fig. 5b, left) – is instructive. The calculated geometry of the halogen bond in [**1·10**] (*r*(N···I) = 2.875 Å; ∠C–I···N = 178.5°) reveals an interaction that is marginally shorter and more linear than those in [**9b·9b**]. The geometry of the [**1·10**] complex, together with the calculated enthalpy of dimerisation at 298 K (–17.9 kJ) – more than half the total for [**9b·9b**], suggested that a slight structural mismatch may be present in the [**9b·9b**] complex, preventing it from taking full advantage of both halogen bonds.

**Fig. 6:**
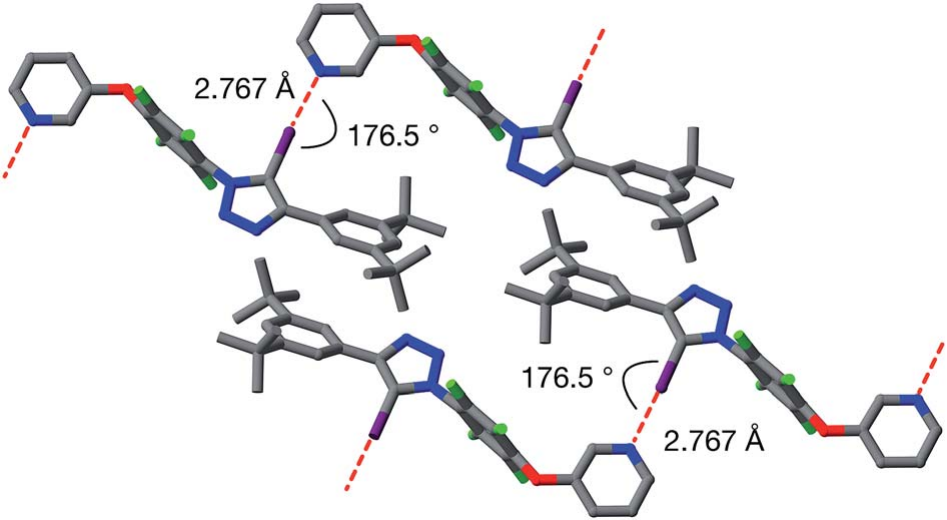
Solid state structure of **9a** determined from single crystal X-ray diffraction data. Potential halogen bonds are marked in red (*r*(N···I) = 2.767 Å; ∠C–I···N = 176.5°). Atom colouring: C atoms = grey, N atoms = blue, O atoms = red, F atoms = light green, I atoms = purple. Hydrogen atoms are omitted for clarity.

Single crystals of **9a**, suitable for analysis by X-ray diffraction, were grown by slow evaporation of a solution of **9a** in toluene. The solid-state structure of **9a** (Fig. 6) reveals antiparallel chains of molecules connected by halogen bonds between the pyridine of one molecule and the iodotriazole of the next. The geometry of these close contacts between the pyridine ring nitrogen atoms and the iodotriazole rings are suggestive of strong halogen bonds – *r*(N···I) = 2.767 Å; ∠C–I···N = 176.5°.

Despite the absence of homodimers in the solid state structure of **9a**, we wished to characterise the stability of the [**9a·9a**] complex in solution. Accordingly, we performed a dilution experiment in order to assess the stability of the [**9a·9a**] dimer in C_6_D_6_ solution. From a starting concentration of 200 mM, progressive dilution of a solution of **9a** resulted in chemical shift changes in the ^1^H NMR spectrum for the resonances associated with the pyridine ring. These chemical shift changes were fitted^28^ (see ESI† for details) to a dimerisation binding model, affording a stability constant for the [**9a·9a**] dimer in C_6_D_6_ of 3.4 ± 0.7 M^–1^. This value was disappointingly low and indicated that the [**9a·9a**] dimer benefits from little, if any, cooperativity^31^ arising from the connection of the halogen bond donor and acceptor within the same molecule.

**Fig. 7:**
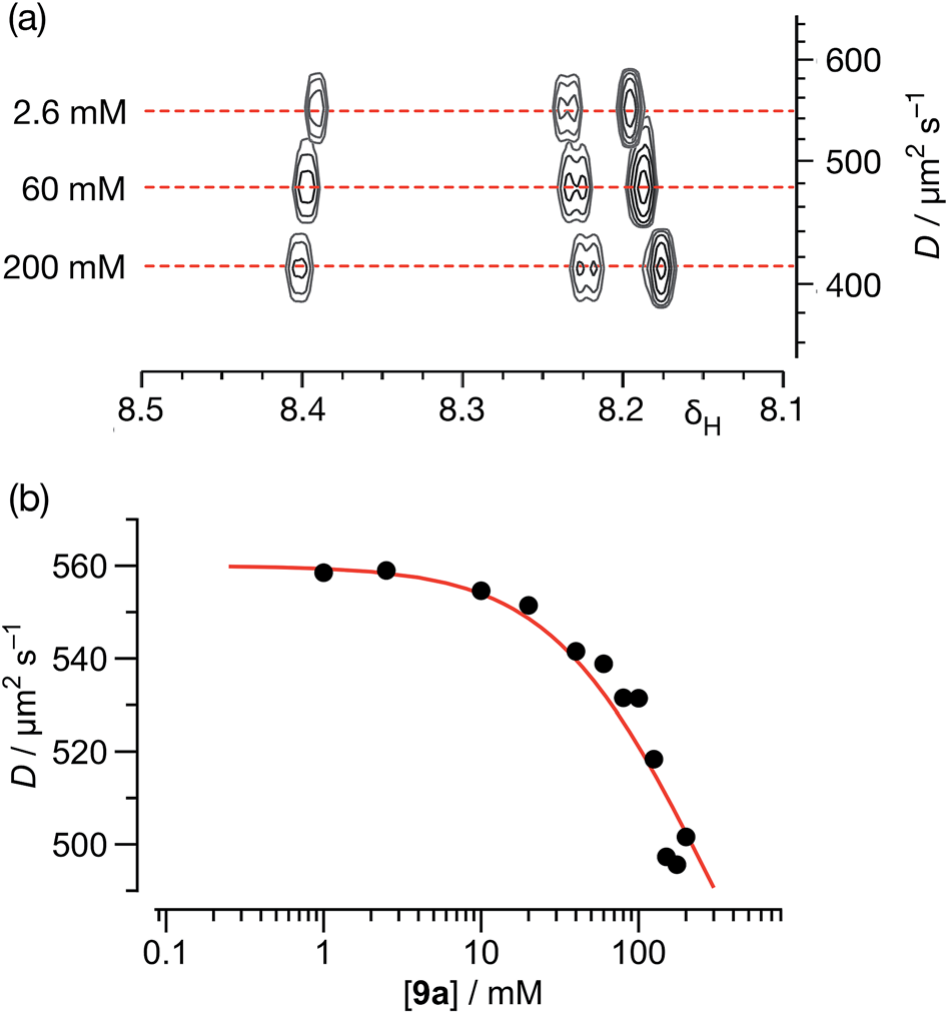
(a) A series of 500.1 MHz ^1^H DOSY NMR experiments, performed on solutions of **9a** in C_6_D_6_ at 298 K, illustrating the decrease in the measured diffusion coefficient (*D*) for **9a** with increasing concentration. (b) Variation of diffusion coefficients (*D*), corrected for viscosity, with respect to the concentration of **9a** in C_6_D_6_ solution at 298 K. The red line shows the best fit of these data to a monomer ⇆ dimer equilibrium model with a *K*_a_ for the [**9a·9a**] homodimer of 2.1 ± 0.4 M^–1^ (see ESI† for details).

In order to characterise the association of **9a** in C_6_D_6_ solution further, we turned to DOSY NMR experiments to assess the nature of the assembly formed. A series of DOSY experiments were performed (Fig. 7a) on sample **9a** in C_6_D_6_, at concentrations ranging from 200 mM down to 1.0 mM. The diffusion coefficients of **9a** and that of the solvent were measured at each concentration. The observed variations of the diffusion coefficient of the solvent were interpreted as a variation in the viscosity of the sample.

Using this data, the diffusion coefficients of solute **9a** were corrected for viscosity changes using the diffusion coefficient for the solvent (C_6_D_6_) measured on the same samples. The variation of these corrected diffusion coefficients for **9a** across the concentration range studied were then fitted (Fig. 7b, see ESI† for further details) to a simple dimerisation model, using the model^32^ of Morris and co-workers for estimating the diffusion of the dimer. The results of this fitting procedure confirm the presence of dimer [**9a·9a**] in solution, and the estimated value of the stability constant for [**9a·9a**] at 298 K – 2.1 ± 0.4 M^–1^ – was in good agreement with that obtained using the more conventional NMR titration method.

## Conclusions

We have demonstrated that 1,4-diaryl-5-iodo-1,2,3-triazoles possess XB properties that make them reliable, neutral XB donors in organic solvents, able to interact with pyridine XB acceptors with efficiencies similar to those displayed by the iconic XB donor iodopentafluorobenzene. The synthetic versatility of these molecular scaffolds allowed the facile construction a self-complementary molecular module, incorporating both an XB donor and an XB acceptor, that was capable of forming a homodimer through the formation of two neutral XB interactions. The stability of this dimeric assembly was evaluated by means of DFT calculations and in C_6_D_6_ solutions using ^1^H NMR and DOSY experiments. The results of these investigations showed that, despite the increased stability of the dimeric assembly, full exploitation of the chelate effect could not be achieved as a result of a partial structural mismatch between the two monomeric units. Nevertheless, the application of chelate cooperativity represents a valid strategy to reinforce XB interactions between two neutral partners in solution and further studies directed towards the optimisation of the monomer design are currently underway in our laboratory.
