# Supplementary material for: Neutral iodotriazoles as scaffolds for stable halogen-bonded assemblies in solution
Source: Chem Sci. 2016 Jun 23;7(10):6422–8. doi: 10.1039/c6sc01974a (PMC5355977; doi:10.1039/c6sc01974a)
Supplement: Supplementary file 1 [file SC-007-C6SC01974A-s001.pdf]

Electronic Supporting Information

Neutral iodotriazoles as scaffolds for stable halogen-bonded assemblies in solution

Leonardo Maugeri,[a] Julia Asencio-Hernández,[b] Tomáš Lébl,[a] David B. Cordes,[a] Alexandra M.Z. Slawin,[a] Marc-André Delsuc[b] and Douglas Philp[a]

[a]:*School of Chemistry and EaStChem, University of St Andrews, North Haugh, St Andrews, Fife KY16 9ST (UK).*

[b]:*Institute de Génétique et de Biologie Moleculaire et Cellulaire INSERM, U596, CNRS, UMR-7104, Université de Strasbourg, 1 rue Laurent Fries, 67404 Illkirch-Graffenstanden (France).*

.

Table of contents

S1. Reagents and General Equipment 3

S2. Synthetic Procedures 4

Synthesis of 3-pentyloxy-pyridine (**8**) 4

Synthesis of 1,3-di-*tert*-butyl-5-(iodoethynyl)benzene (**S1**) 4

Synthesis of 4-azido-2,3,5,6-tetrafluoro-1,1'-biphenyl (**S3**) 5

Syntheses of 5-iodo-1,2,3-triazoles 6

Synthesis of 3-(4-(4-(3,5-Di-tert-butylphenyl)-5-iodo-1H-1,2,3-triazol-1-yl)-2,3,5,6-tetrafluorophenoxy)pyridine (**9a**) 7

S3. XB association constant determination 9

S4. DFT calculations 13

S5. X-Ray crystal structures of iodotriazoles **7** and **S4** 14

S6. References 15

S7. Crystallographic Informations 17

S8. NMR spectra 44

S1. Reagents and General Equipment

All reagents were purchased from commercial sources (Alfa Aesar, Apollo Scientific Ltd., Fisher Scientific UK Ltd., Fluorochem UK Ltd., TCI UK Ltd. and Sigma–Aldrich Company Ltd) unless stated otherwise, and used without further purification.

Dry solvents were obtained by means of a MBBRAUN MB SPS-800™ purification system.

Flash column chromatography were performed using Geduran® Si60 (40-63 μM, Merck, Germany) as the stationary phase and thin layer chromatography was performed on pre-coated silica gel-plates (0.25 mm thick, $60F_{254}$, Merck, Germany) and observed under UV light irradiation.

^{1}H, ^{13}C, ^{19}F NMR spectroscopic data was acquired using either a Bruker Avance (500 MHz) or a Bruker Avance III (500 MHz) spectrometer, at a constant temperature of 25 ºC. ^{1}H and ^{13}C chemical shifts are reported in parts per million (ppm) from high to low field and referenced to the literature values for chemical shift of the residual non-deuterated solvent, with respect to tetramethylsilane. ^{19}F NMR chemical shifts are also reported in parts per million and are referenced to $CFCl_3$ (0.00 ppm).

^{1}H–^{15}N HMBC NMR experiments were performed on a Bruker Avance III HD 700 MHz spectrometer.

All melting points were measured using a Stuart SMP30 melting point apparatus.

Mass spectra were recorded on a Micromass GCT spectrometer for chemical ionisation (CI) using isobutene as the ionising gas. Electron spray ionisation (ESI) spectra were performed on a Micromass LCT spectrometer operating in positive or negative mode, *m/z* values are reported in Daltons.

S2. Synthetic Procedures

Iodoethynyl benzene,[1] *N*-iodo morpholine,[1] pentafluorophenylazide,[2] 1,3-di-*tert*-butyl-5-ethynylbenzene[3] were prepared according to previously described literature procedures.

Synthesis of 3-pentyloxy-pyridine (8)

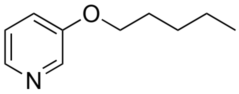

Sodium hydride (15.4 mmol, 60% dispersion in mineral oil) was added to a solution of pentan-1-ol (1.36 g, 15.4 mmol) in DMF (30.0 mL), and the suspension thus obtained was stirred at room temperature for 1 hour. Subsequently, 3-fluoro-pyridine (1.50 g, 15.4 mmol) was added and the mixture was stirred overnight at room temperature. The reaction was quenched by addition of water and extracted with ethyl acetate. The combined organic phases were washed with LiCl saturated solution, dried over $MgSO_4$, filtered and concentrated *in vacuo*. Purification of the product was achieved by column chromatography of the crude mixture (CH_2Cl_2: EtOAc 9:1) to afford of 3-(pentyloxy)-pyridine **8** (1.80 g, 10.8 mmol, 70%) as a pale yellow oil.

^{1}H NMR (500.1 MHz; $CDCl_3$): δ 8.29 (dd, J = 2.7 Hz and J = 0.5 Hz, 1H), 8.18 (dd, J = 4.3 Hz and J = 1.6 Hz, 1H), 7.19–7.14 (m, 2H), 3.97 (t, J = 6.6 Hz, 2H), 1.81–1.75 (m, 2H), 1.46–1.33 (m, 4H), 0.92 (t, J = 7.2 Hz, 3H). ^{13}C NMR (125.7 MHz; $CDCl_3$): δ 155.3, 142.0, 138.1, 123.9, 121.1, 68.4, 28.9, 28.2, 22.5, 14.1. HRMS pESI $[M+H]^+$ calcd. for $C_{10}H_{16}NO$ 162.1232; found 162.1221.

Synthesis of 1,3-di-*tert*-butyl-5-(iodoethynyl)benzene (S1)

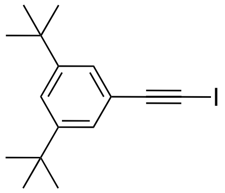

1,3-Di-*tert*-butyl-5-ethynylbenzene (2.11 g, 9.87 mmol) was dissolved in THF (24.0 mL), CuI (0.92 mg, 0.48 mmol) and *N*-iodo morpholine (3.75 g, 11.0 mmol) were added and the resulting mixture was stirred at room temperature until a white precipitate formed (approximately 3 hours). The reaction mixture was then filtered through a pad of neutral alumina and the solid phase was washed with CH_2Cl_2. The collected organic fractions were concentrated *in vacuo* to afford **S1** as a colourless solid (2.99 g, 8.78 mmol, 89%).

M.p. 106.3–108.7 °C. ^{1}H NMR (400.1 MHz; $CDCl_3$): δ 7.39 (t, *J* = 1.9 Hz, 1H), 7.30 (d, *J* = 1.9 Hz, 2H), 1.31 (s, 18H). ^{13}C NMR (100.6 MHz; $CDCl_3$): δ 150.8, 126.5, 123.3, 122.4, 95.3, 34.8, 31.3, 4.1. HRMS CI+ $[M]^+$ calcd. for $C_{16}H_{22}I$, 341.0766; found 341.0763.

Synthesis of 4-azido-2,3,5,6-tetrafluoro-1,1'-biphenyl (S3)

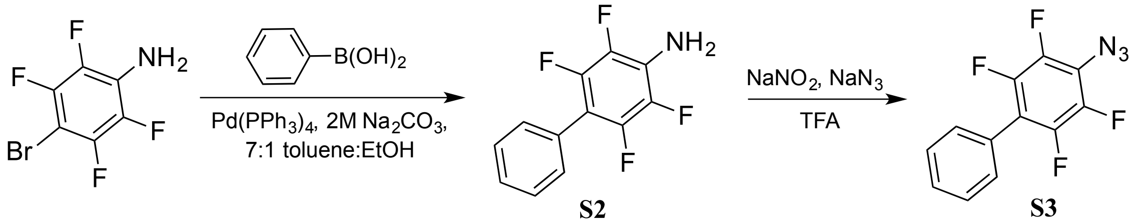

S2 **S3**

Scheme S1 Synthesis of -azido-2,3,5,6-tetrafluoro-1,1'-biphenyl **S3**.

2,3,5,6-Tetrafluoro-[1,1'-biphenyl]-4-amine (S2). 4-Bromo-2,3,5,6-tetrafluoro aniline (3.00 g, 12.3 mmol) and phenylboronic acid (4.50 g, 36.9 mmol) were dissolved into a 7:1 toluene:ethanol mixture (48.0 mL). An aqueous Na_2CO_3 solution (13.0 mL, 2 M) was added to the mixture and the suspension thus obtained was degassed by bubbling Ar through the reaction flask for 30 minutes. $Pd(PPh_3)_4$ (0.85 g, 0.74 mmol) was added and the reaction mixture was heated overnight to 90 °C. After this time, the reaction was quenched by adding water, and the organic material was extracted with ethyl acetate. The organic phase was washed with brine, dried over $MgSO_4$ and concentrated *in vacuo*. Purification of the crude mixture by column chromatography (1:1 hexane: EtOAc) afforded product **S2** (2.66 g, 11.0 mmol, 89%) as a colourless solid.

M.p.: 92.3–94.0 °C. ^{1}H NMR (500.1 MHz; $CDCl_3$): δ 7.48–7.39 (m, 5H), 4.04 (s, 2H). ^{19}F NMR (470.5 MHz; $CDCl_3$): δ −146.67–−146.75 (m, 2F), −162.16–−162.24 (m, 2F). ^{13}C NMR (125.7 MHz; $CDCl_3$): δ 145.2–143.0 (m, 1C), 137.9–135.7 (m, 1C), 130.3 (t, *J* = 2.0 Hz), 128.5, 128.3, 128.0, 125.3 (tt, *J* = 14.2 Hz and *J* = 3.9 Hz), 108.3 (t, *J* = 14.2 Hz). HRMS nESI $[M-H]^-$ calcd. for $C_{12}H_6F_4N$ 240.0442; found 240.0443.

4-azido-2,3,5,6-tetrafluoro-1,1'-biphenyl (S3). Aniline **S2** (1.60 g, 6.63 mmol) was dissolved in TFA (16 mL). The solution was cooled to 0 ºC and $NaNO_2$ (685 mg, 9.95 mmol) was slowly added and the mixture was left to react for 1 hour at the same temperature. After this time NaN_3 (689 mg, 10.6 mmol) was added in small portions over a period of 10 minutes and the mixture was left to react at 0 °C for 1 further hour. The reaction mixture was then diluted with ether and transferred into a separating funnel and washed with a saturated Na_2CO_3 solution until complete neutralization

of the acid. The organic phase was then washed with brine, dried over $MgSO_4$ and concentrated *in vacuo* to afford product **S3** as a yellow solid (1.58 g, 5.91 mmol, 89%).

M.p.: 72.3–73.7 °C .^{1}H NMR (500.1 MHz; $CDCl_3$): δ 7.51–7.43 (m). ^{19}F NMR (470.5 MHz; $CDCl_3$): δ −143.7 (dd, *J* = 21.6 Hz and *J* = 9.8 Hz, 2F), −152.2 (dd, *J* = 21.6 Hz and *J* = 9.8 Hz, 2F). ^{13}C NMR (125.7 MHz; $CDCl_3$): δ 145.1–143.0 (m), 140.8 (ddt, *J* = 249.1 Hz, *J* = 17.2 Hz and *J* = 3.9 Hz), 130.1 (t, *J* = 2.0 Hz), 129.2, 128.7, 126.8 (m), 118.75 (tt, *J* = 12.3 Hz and *J* = 3.0 Hz), 116.4 (t, *J* = 17.2 Hz).

HRMS GCTOF $[M+H]^+$ calcd. for $C_{12}H_6F_4N_3$ 268.0498; found 268.0500.

Syntheses of 5-iodo-1,2,3-triazoles

5-Iodo-1-(perfluorophenyl)-4-phenyl-1*H*-1,2,3-triazole (1)

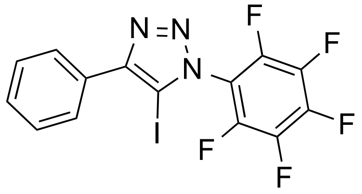

Pentafluorophenylazide (1.00 g, 4.8 mmol) and iodoethynyl benzene (1.09 g, 4.8 mmol) were dissolved in THF (24.0 mL). Copper iodide (0.46 g, 0.24 mmol) and triethylamine (1.3 mL, 9.3 mmol) were added and the mixture was stirred overnight at room temperature. After this time, the reaction was quenched by addition of 4.8 mL of a 10% NH_4OH solution, and the resulting crude concentrated by rotary evaporation. The concentrated crude was then redissolved in ethyl ether and transferred in a separating funnel. The organic phase was washed with water, brine, dried over $MgSO_4$ and concentrated *in vacuo*. The organic residue was triturated with hexane and the product was isolated by filtration, yielding **1** (1.26 g, 2.9 mmol, 60%) as a colourless solid. Crystals suitable for single crystal X-ray diffraction were obtained upon cooling of a saturated toluene solution.

M.p. >170 °C dec.. ^{1}H NMR (500.1 MHz; $CDCl_3$): δ 8.04–8.03 (m, 2H), 7.54–7.45 (m, 3H). ^{19}F NMR (470.5 MHz; $CDCl_3$): δ −142.24–−142.28 (m, 2F), −147.29–−147.39 (m, 1F), −159.14–−159.23 (m, 2F). ^{13}C NMR (125.7 MHz; $CDCl_3$): δ 150.7, 144.9–142.7 (m), 144.7–142.3 (m), 139.2–136.9 (m), 129.4, 129.2, 128.9, 127.6, 112.6–112.4 (m), 80.2.

HRMS pESI $[M+H]^+$ calcd. for $C_{14}H_6F_5IN$ 437.9527; found 437.9522.

4-(3,5-Di-*tert*-butylphenyl)-5-iodo-1-(perfluorophenyl)-1*H*-1,2,3-triazole (7)

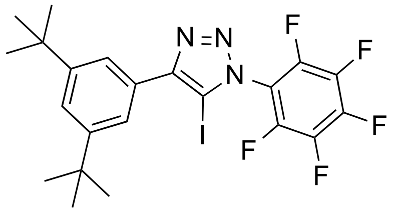

This molecule was prepared using the same procedure used for **1**. Purification of the product was achieved by column chromatography of the worked-up crude (1:1 petroleum ether: CH_2Cl_2), affording iodotriazole **7** as a colourless solid (62%). Crystals suitable for single crystal X-ray diffraction were obtained upon cooling of a saturated toluene solution.

M.p. >155 °C dec.. ^{1}H NMR (500.1 MHz; $CDCl_3$): δ 7.88 (d, J = 1.8 Hz, 2H), 7.52 (t, J = 1.8 Hz, 1H), 1.40 (s, 18H). ^{19}F NMR (470.5 MHz; $CDCl_3$): δ −142.22–−142.27 (m, 2F), −147.57 (t, J = 21.3 Hz, 1F), −159.24–−159.37 (m, 2F). ^{13}C NMR (125.7 MHz; $CDCl_3$): δ 151.5, 151.4, 145.0–142.7 (m), 144.6–142.3 (m), 139.2–136.9 (m), 128.2, 123.4, 122.0, 112.8–112.5 (m), 80.1, 35.3, 31.6. HRMS+ pESI (*m/z*) [M+Na]$^+$ calcd. for $C_{22}H_{21}F_5IN_3Na$ 572.0593; found 572.0576

5-iodo-4-phenyl-1-(2,3,5,6-tetrafluoro-[1,1'-biphenyl]-4-yl)-1*H*-1,2,3-triazole (S4)

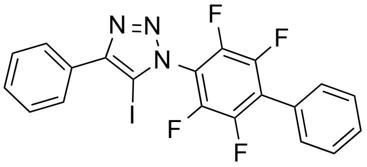

This molecule was prepared using the same procedure used for **1**. Purification of the product was achieved by column chromatography of the worked-up crude (5:1 petroleum ether:EtOAc) followed by crystallization in hot acetonitrile, affording iodotriazole **S4** as a colourless solid (70%). Crystals suitable for single crystal X-ray diffraction were obtained upon cooling of a saturated toluene solution.

M.p.: > 180 °C dec.. ^{1}H NMR (500.1 MHz; $CDCl_3$): δ 8.08–8.06 (m, 2H), 7.59–7.52 (m, 7H), 7.49–7.45 (m, 1H). ^{19}F NMR (470.5 MHz; $CDCl_3$): δ −141.27 (m, 2F), −143.76 (m, 2F). ^{13}C NMR (125.7 MHz; $CDCl_3$): δ 150.5, 145.2–143.0 (m), 144.4–142.3 (m), 130.2 (t, J = 1.9 Hz), 130.1, 129.4, 129.3, 129.0, 128.9, 127.6, 126.2, 124.3 (t, J = 16.6 Hz), 115.3–115.1 (m), 80.1. HRMS pESI [M+H]$^+$ calcd. for $C_{20}H_{11}F_4IN_3$ 495.9934; found 495.9918.

Synthesis of 3-(4-(4-(3,5-Di-tert-butylphenyl)-5-iodo-1H-1,2,3-triazol-1-yl)-2,3,5,6-tetrafluorophenoxy)pyridine (9a)

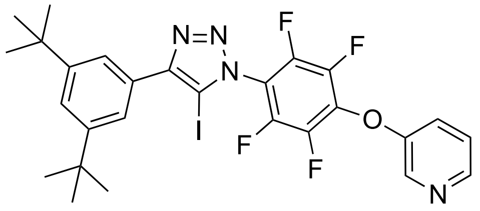

In a dry flask sodium hydride (1.82 mmol, 60% dispersion in mineral oil) was added to solution of 3-hydroxy-pyridine (173 mg, 1.82 mmol) in DMF (5 mL). The resulting suspension was stirred at room temperature for 1 hour. After this time, iodotriazole **7** (1.00 g, 1.82 mmol) was added and the mixture was stirred at room temperature overnight. The reaction was quenched by the addition of water and transferred into a separating funnel. The aqueous phase was extracted with EtOAc for three times, the organic extracts were combined and washed with LiCl saturated solution, dried over $MgSO_4$ and concentrated under reduced pressure. The resulting crude was purified by column chromatography (5:1 DCM:Et_2O), yielding product **9a** as a colourless solid (864 mg, 1.38 mmol, 76%). Crystals suitable for single crystal X-ray diffraction were obtained upon cooling of a saturated toluene solution.

M.p. >160 °C dec.. ^1H NMR (500.1 MHz; $CDCl_3$): δ 8.53 (d, J = 2.7 Hz, 1H), 8.48 (dd, J = 1.9 Hz and J = 4.5 Hz, 1H), 7.90 (d, J = 1.7 Hz, 2H), 7.52 (t, J = 1.7 Hz, 1H), 7.40 (m, 1H), 7.36 (dd, J = 8.3 Hz and J = 4.5 Hz, 1H), 1.40 (s, 18H). ^{19}F NMR (470.5 MHz; $CDCl_3$): δ −142.31–−142.39 (m, 2F), −151.59–−151.66 (m, 2F). ^{13}C NMR (125.7 MHz; $CDCl_3$): δ 153.2, 151.4, 151.3, 145.9, 143.9 (ddt, J = 258.7 Hz, J = 13.1 Hz and J = 3.7 Hz), 145.9, 142.6–140.4, 138.8, 135.9 (tt, J = 12.7 Hz and J = 2.7 Hz), 128.1, 123.3, 123.1, 121.9, 113.4 (t, J = 14.5 Hz), 80.1, 35.1, 31.5.

HRMS+ pESI (m/z) $[M+Na]^+$ calcd. for $C_{27}H_{25}OF_4IN_4Na$ 647.0901; found 647.0892.

S3. XB association constant determination

Association constants for 1:1 complexes were determined[4] by NMR spectroscopy using standard methods. The stability of complexes [**1•3**], [**2•3**], [**2•8**] and [**7•8**] were determined from the fitting of either ^{1}H and/or ^{15}N chemical shift changes to a 1:1 binding model using the WinEQNMR[5] program. Data for this fitting process were generated by titration of the XB donor into a 20 mM solution of the appropriate XB acceptor in d_8-toluene at 293 K and a 700.1 MHz ^{1}H-^{15}N HMBC correlation experiment was performed on the sample at each concentration step, allowing the extraction of both ^{1}H and ^{15}N chemical shifts. The best fit of the appropriate binding models to the ^{15}N NMR data are shown in **Figure S1**.

The stability of dimer [**9a•9a**] was determined from fitting of ^{1}H chemical shift changes to dimerization model using the WinEQNMR[5] program. Data for this fitting process were generated by dilution of a solution of compound **9a** in C_6D_6 at 298 K from 200 mM to 2.6 mM in 11 steps. At each concentration, a 500.1 MHz ^{1}H NMR spectrum was recorded on the sample allowing the extraction of ^{1}H chemical shifts. The best fit of the appropriate dimerisation model to the ^{1}H NMR data afforded a K_a of 3.4 ± 0.6 M^{-1} for the [**9a•9a**] dimer and is shown in **Figure S2**.

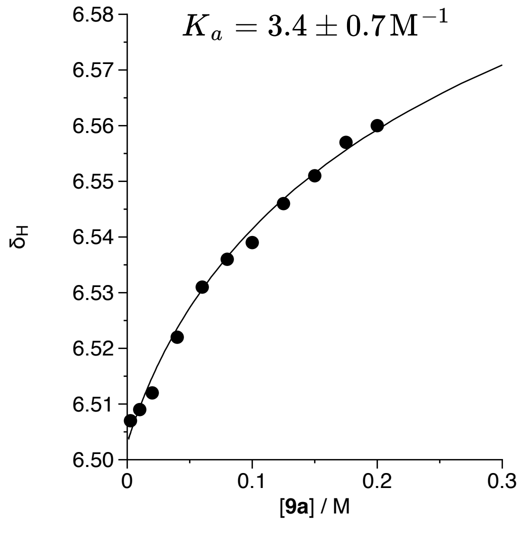

Figure S2. Variation of the ^{1}H chemical shift of an aromatic probe proton in compound **9a** as a function the concentration. The solid line represents the best fit of a dimerisation model to the data.

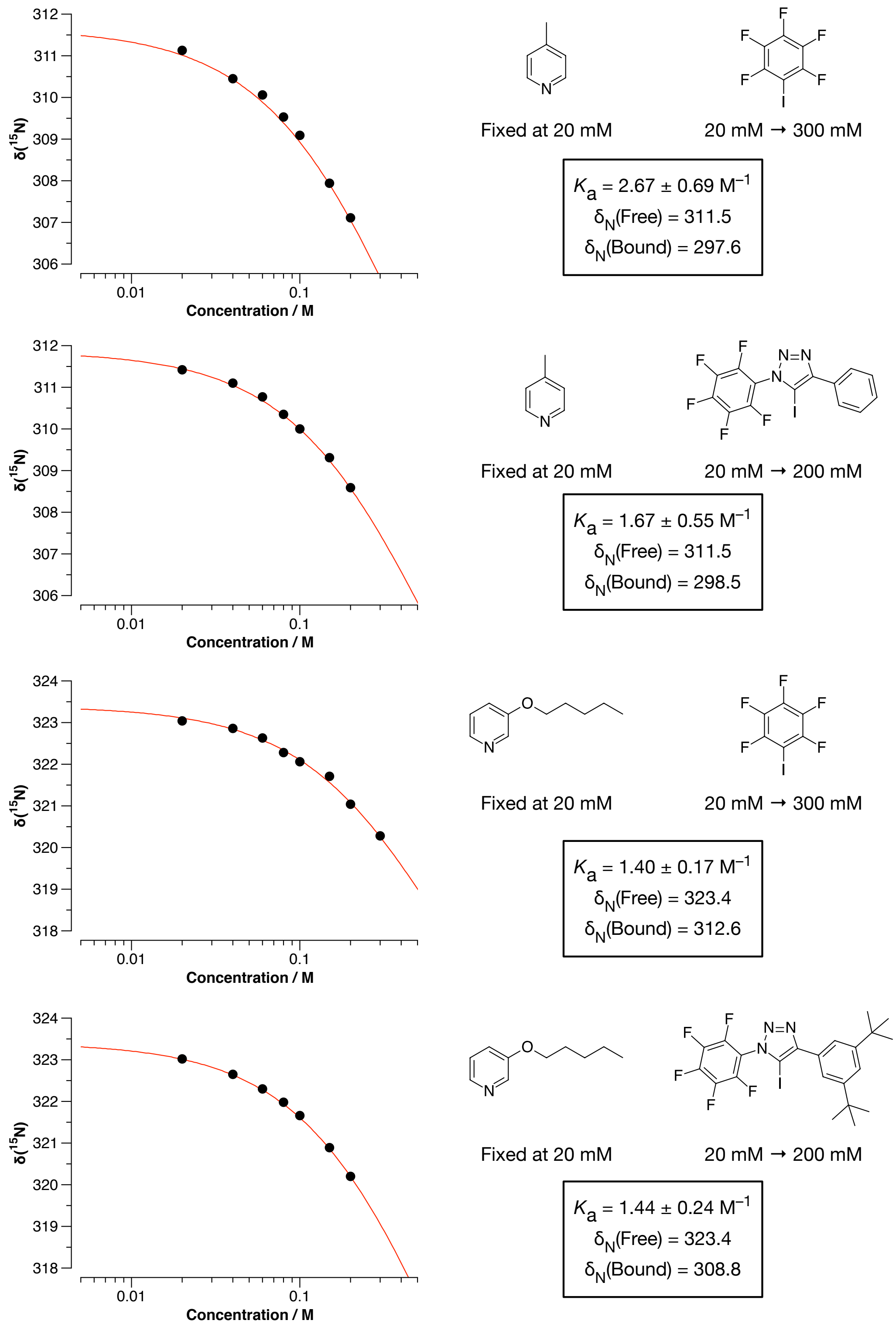

Figure S1. The stability of complexes [**1•3**], [**2•3**], [**2•8**] and [**7•8**] determined from the fitting of ^{15}N chemical shift changes to a 1:1 binding model using the WinEQNMR[5] program. Data for this fitting process were generated by titration of the XB donor into a 20 mM solution of the appropriate XB acceptor in d_8-toluene at 293 K and a 700.1 MHz The best fit of the appropriate binding models to the ^{15}N NMR data (black circles) are as the solid red lines.

A series of DOSY NMR experiments were performed on the same set of samples of **9a** in C_6D_6, allowing the measurement of the diffusion coefficient of **9a** (D(**9a**)) and C_6D_6 ($D(C_6D_6)$).
The values of D(**9a**) were then corrected for changes in solvent viscosity using the diffusion coefficients measured from C_6D_6, thus generating a new set of data, namely D_c(**9a**) (**Figure S3**). These data are listed in **Table S1**.

Table S1. Diffusion coefficients of **9a** and C_6D_6 for each concentrations of **9a** measured by 500.1 MHz DOSY ^{1}H NMR spectroscopy at 298 K.

[**9a**] / mM	D(**9a**) / μm²s⁻¹	$D(C_6D_6)$ / μm²s⁻¹	D_c(**9a**)* / μm²s⁻¹
1	558.5	1763	558.5
2.6	565.0	1782	558.9
10	554.6	1763	554.6
20	548.0	1752	551.4
40	518.8	1689	541.5
60	498.8	1632	538.8
80	489.5	1624	531.5
100	486.5	1614	531.4
125	462.8	1574	518.4
150	437.5	1551	497.3
175	421.4	1499	495.6
200	418.8	1472	501.6

*: D_c(**9a**) = D(**9a**) × 1763 / $D(C_6D_6)$

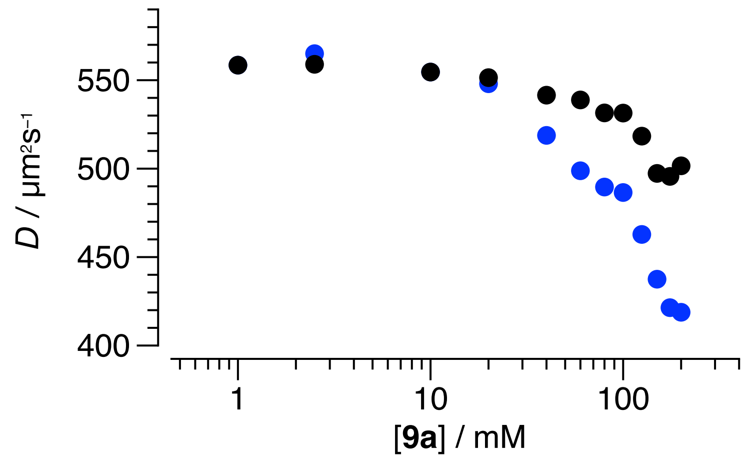

Figure S3. Plot of the diffusion coefficients of **9a**, D(**9a**) (•) and D_c(**9a**) (•), as a function of the concentration of **9a**.

The variation of the corrected diffusion coefficient, D_c(**9a**), was fitted against a simple dimerisation model, using a variant of the model[6] of Morris *et al.* to estimate the diffusion coefficient of the [**9a**•**9a**] dimer.

The dimerization model applied is described by the following set of equations, where M = **9a**:

$$2M \rightleftarrows M_2$$

with mass action law

$$K_a = \frac{[M]}{[M]^2}$$

and mass conservation

$$[M] + 2[M_2] = M_0$$

simplifying, we get

$$[M_2] = 0.5(M_0 - [M])$$

$$[M]^2 K_a + 1/2[M] - 1/2M_0 = 0$$

Solving the second order equation:

$$\Delta = 1/4 + 2K_a M_0$$

$$[M] = \frac{-1/2 + \sqrt{\Delta}}{2K_a}$$

Non-linear regression of this model to the diffusion coefficient data (**Figure S4**) afforded a value of $2.1 \pm 0.4\ M^{-1}$ at 298 K in C_6D_6 for the stability constant (K_a) for [**9a•9a**].

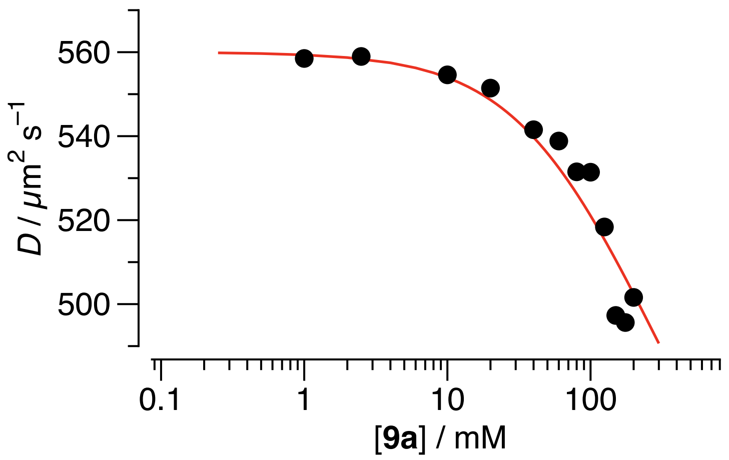

Figure S4. The red line shows the best fit of these data to a monomer ⇆ dimer equilibrium model with a K_a for the [**9a•9a**] homodimer of $2.1 \pm 0.4\ M^{-1}$.

S4. DFT calculations

All calculations were performed using Gaussian09[7] suite of programs – either revision A.02 or revision D.01 was used in all calculations. Calculation were performed using TPSSh[8] functional and the def2-TZVP basis set[9] of Weigend and Ahlrichs. This basis set is not standard in Gaussian09 and was introduced using the `GenECP` keyword using an appropriately-formatted input block for the basis set generated from data obtained from the Basis Set Exchange[10] (https://bse.pnl.gov/bse/portal). An effective core potential on iodine,[11] which replaces 28 valence electrons on each iodine atom in the structure was used in all calculations. The basis set is all-electron for all other elements.

All geometries were optimized fully in internal (keyword: `opt`) or cartesian (keyword: `opt=cartesian`) coordinates using the default optimisation protocols within Gaussian09. Stationary points were characterised by means of a vibrational analysis (keyword: `freq`) and zero-point energy corrections and other thermodynamic parameters, used in the calculation of interactions energies, were derived[12] from this analysis. Population analyses using the Natural Bond Orbital (NBO) method were performed using the NBO6 program[13,14] in a two-stage procedure. The input for NBO6 was generated using Gaussian09 (keyword: `population=nboread`) and the .47 file generated by this calculation was then edited and processed using the `gennbo` script provided with the NBO6 distribution. All analyses used the 8-byte integer version of NBO6 dated March 2015 compiled from source using *gfortran* (Version 4.4.7).

The basis set superposition error was calculated using the counterpoise method[15] as implemented within Gaussian09 (keyword: `counterpoise = 2`).

Halogen bonds were visualized using the NCIPLOT[16] program. SCF densities written as an extended wavefunction file from Gaussian09 (rev. D.01) and NCIPLOT used to generate Gaussian cube files from which isosurfaces could be visualised using VMD[17] with $s = 0.5$ and colours (blue (attractive) to red (repulsive)) mapped on to $-0.05 < \rho < 0.05$.

S5. X-Ray crystal structures of iodotriazoles 7 and S4

Suitable crystals of iodotriazoles **7** and **S4** were obtained by slow evaporation of a saturated toluene solution and their structures are reported in **Figure S5a** and **S5b** respectively. Similarly to iodotriazole **1**, iodotriazole **S4** crystallises in antiparallel chains held together by the short XB contact between the iodine atom of one iodotriazole XB donor and the 3-nitrogen atom of the neighbouring iodotriazole unit – *r*(N•••I) = 2.939 Å, ∠(C–I•••N) = 169.3º.

On the other hand, the crystal structure of iodotriazole **7**, originally synthesised to provide a more soluble version of iodotriazole **1**, shows no XB contacts in the solid state (**Figure S5b**). Evidently, the substitution of two hydrogen atoms with two bulky *tert*-butyl groups affects negatively the capability of this class of Lewis acidic molecules to perform as XB donors in the solid state.

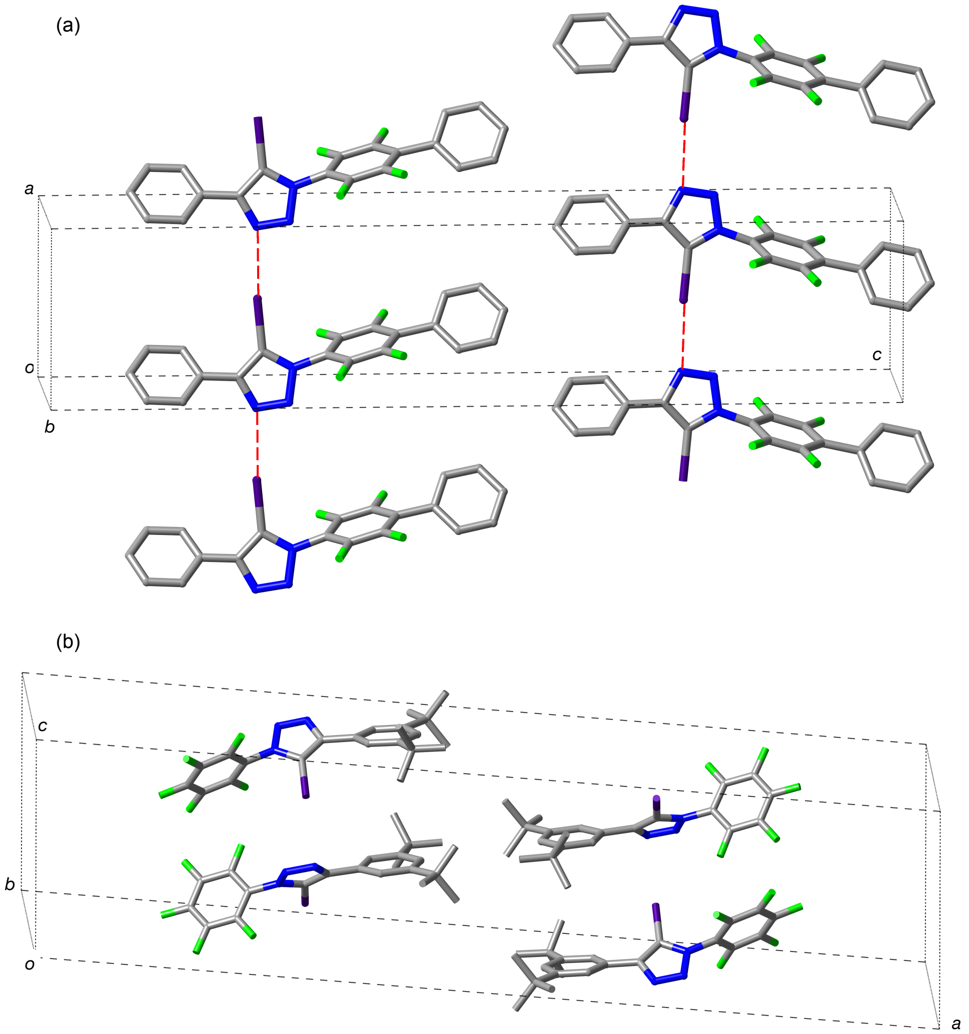

Figure S5. (a) X-Ray crystal structure of iodotriazole **S4**: the substitution of the 4-fluorine atom with a phenyl group does not prevent the formation of antiparallel polymeric chains of iodotriazole units held together by a short XB contact (*r*(N•••I) = 2.94 Å, ∠(C–I•••N) = 169º). (b) X-Ray crystal structure of iodotriazole **7**: the introduction of two bulky *tert*-butyl groups in place of two hydrogen atoms prevents the formation of any XB-based crystal packing.

S6. References

[1] J. E. Hein, J. C. Tripp, L. B. Krasnova, K. B. Sharpless, V. V. Fokin, *Angew. Chem. Int. Ed.* **2009**, *48*, 8018–8021.

[2] K. Kanakarajan, K. Haider, A. W. Czarnich, *Synthesis (Stuttg).* **1988**, 566–568.

[3] D. Philp, V. Gramlich, P. Seiler, F. Diederich, *J. Chem. Soc. Perkin Trans. 2* **1995**, 875–886.

[4] P. Thordarson, *Chem. Soc. Rev.* **2011**, *40*, 1305–1323.

[5] M. J. Hynes, *J. Chem. Soc. Dalt. Trans.* **1993**, 311–312.

[6] R. Evans, Z. Deng, A. K. Rogerson, A. S. McLachlan, J. J. Richards, M. Nilsson, G. A. Morris, *Angew. Chem. Int. Ed.* **2013**, *52*, 3199–3202.

[7] Gaussian 09, Revision A.02 or D.01, M. J. Frisch, G. W. Trucks, H. B. Schlegel, G. E. Scuseria, M. A. Robb, J. R. Cheeseman, G. Scalmani, V. Barone, B. Mennucci, G. A. Petersson, H. Nakatsuji, M. Caricato, X. Li, H. P. Hratchian, A. F. Izmaylov, J. Bloino, G. Zheng, J. L. Sonnenberg, M. Hada, M. Ehara, K. Toyota, R. Fukuda, J. Hasegawa, M. Ishida, T. Nakajima, Y. Honda, O. Kitao, H. Nakai, T. Vreven, J. A. Montgomery, Jr., J. E. Peralta, F. Ogliaro, M. Bearpark, J. J. Heyd, E. Brothers, K. N. Kudin, V. N. Staroverov, R. Kobayashi, J. Normand, K. Raghavachari, A. Rendell, J. C. Burant, S. S. Iyengar, J. Tomasi, M. Cossi, N. Rega, J. M. Millam, M. Klene, J. E. Knox, J. B. Cross, V. Bakken, C. Adamo, J. Jaramillo, R. Gomperts, R. E. Stratmann, O. Yazyev, A. J. Austin, R. Cammi, C. Pomelli, J. W. Ochterski, R. L. Martin, K. Morokuma, V. G. Zakrzewski, G. A. Voth, P. Salvador, J. J. Dannenberg, S. Dapprich, A. D. Daniels, Ö. Farkas, J. B. Foresman, J. V. Ortiz, J. Cioslowski, and D. J. Fox, Gaussian, Inc., Wallingford CT, 2009.

[8] J. Tao, J. P. Perdew, V. N. Staroverov, G. E. Scuseria, *Phys. Rev. Lett.* **2003**, *91*, 146401.

[9] F. Weigend, R. Ahlrichs, *Phys. Chem. Chem. Phys.* **2005**, *7*, 3297–3305.

[10] K. L. Schuchardt, B. T. Didier, T. Elsethagen, L. Sun, V. Gurumoorthi, J. Chase, J. Li, T. L. Windus, *J. Chem. Inf. Model.* **2007**, *47*, 1045–1052.

[11] K. A. Peterson, *J. Chem. Phys.* **2003**, *119*, 11113–11123.

[12] Ochterski, J. W. Thermochemistry in Gaussian; Gaussian Inc.: Wallingford, CT, 2000.

[13] E. D. Glendening, C. R. Landis, F. Weinhold, *J. Comput. Chem.* **2013**, *34*, 1429–1437.

[14] E. D. Glendening, J, K. Badenhoop, A. E. Reed, J. E. Carpenter, J. A. Bohmann, C. M. Morales, C. R. Landis, and F. Weinhold, Theoretical Chemistry Institute, University of Wisconsin, Madison (2013).

[15] S. F. Boys, F. Bernardi, *Mol. Phys.* **1970**, *19*, 553–566.

[16] E. R. Johnson, S. Keinan, P. Mori Sánchez, J. Contreras García, A. J. Cohen, W. Yang, *J. Am. Chem. Soc.* **2010**, *132*, 6498–6506.

[17] Humphrey, W., Dalke, A. and Schulten, K., *J. Molec. Graphics,* 1996, 14, 33-38. http://www.ks.uiuc.edu/Research/vmd/

S7. Crystallographic Informations

The CCDC numbers for compounds **1**, **7**, **9a** and **S4** are 1442418-1442421.

5-Iodo-1-(perfluorophenyl)-4-phenyl-1*H*-1,2,3-triazole (1)

Data Collection

A colorless prism crystal of $C_{14}H_5F_5IN_3$ having approximate dimensions of 0.200 × 0.050 × 0.050 mm was mounted in a loop. All measurements were made on a Rigaku XtaLAB P100 diffractometer Mo-Kα radiation.

Cell constants and an orientation matrix for data collection corresponded to a primitive monoclinic cell with dimensions:

a = 25.720(2) Å

b = 7.5763(7) Å   β = 92.727(3)º

c = 7.2135(7) Å

V = 1404.1(2) $Å^3$

For Z = 4 and F.W. = 437.11, the calculated density is 2.068 g/cm^3. The reflection conditions of:

h0l: l = 2n

0k0: k = 2n

uniquely determine the space group to be:

$P2_1/c$ (#14)

The data were collected at a temperature of −100 ± 1ºC to a maximum 2θ value of 50.7º.

Data Reduction

Of the 30582 reflections were collected, where 2577 were unique (R_{int} = 0.0473); equivalent reflections were merged. Data were collected and processed using CrystalClear (Rigaku).[1]

The linear absorption coefficient, μ, for Mo-Kα radiation is 23.398 cm^{-1}. An empirical absorption correction was applied which resulted in transmission factors ranging from 0.705 to 0.890. The data were corrected for Lorentz and polarization effects.

Structure Solution and Refinement

The structure was solved by heavy-atom Patterson methods[2] and expanded using Fourier techniques. The non-hydrogen atoms were refined anisotropically. Hydrogen atoms were refined using the riding model. The final cycle of full-matrix least-squares refinement[3] on F^2 was based on 2577 observed reflections and 208 variable parameters and converged (largest parameter shift was 0.00 times its esd) with unweighted and weighted agreement factors of:

$$R1 = \Sigma \, ||Fo| - |Fc|| \, / \, \Sigma \, |Fo| = 0.0170$$

$$wR2 = [\Sigma \, (w \, (Fo^2 - Fc^2)^2) / \Sigma \, w(Fo^2)^2]^{½} = 0.0495$$

The goodness of fit[4] was 1.08. Unit weights were used. Plots of $\Sigma \, w(|Fo| - |Fc|)^2$ versus |Fo|, reflection order in data collection, sin θ/λ and various classes of indices showed no unusual trends. The maximum and minimum peaks on the final difference Fourier map corresponded to 0.51 and −0.47 $e^-/Å^3$, respectively.

Neutral atom scattering factors were taken from International Tables for Crystallography (IT), Vol. C, Table 6.1.1.4.[5] Anomalous dispersion effects were included in Fcalc;[6] the values for Δf' and Δf" were those of Creagh and McAuley.[7] The values for the mass attenuation coefficients are those of Creagh and Hubbell.[8] All calculations were performed using the CrystalStructure[9] crystallographic software package except for refinement, which was performed using SHELXL-2013.[10]

References

(1) CrystalClear: Rigaku Corporation, 1999. CrystalClear Software User's Guide, Molecular Structure Corporation, (c) 2000. J. W. Pflugrath (1999) Acta Cryst. D55, 1718-1725.

(2) PATTY: Beurskens, P.T., Admiraal, G., Behm, H., Beurskens, G., Smits, J.M.M. and Smykalla, C. (1991). Z. f. Kristallogr. Suppl.4, p.99.

(3) Least Squares function minimized: (SHELXL2013)

$$\Sigma w(F_o^2 - F_c^2)^2$$ where w = Least Squares weights.

(4) Goodness of fit is defined as:

$$[\Sigma w(F_o^2 - F_c^2)^2 / (N_o - N_v)]^{1/2}$$

where: N_o = number of observations
N_v = number of variables

(5) International Tables for Crystallography, Vol.C (1992). Ed. A.J.C. Wilson, Kluwer Academic Publishers, Dordrecht, Netherlands, Table 6.1.1.4, pp. 572.

(6) Ibers, J. A. & Hamilton, W. C.; Acta Crystallogr., 17, 781 (1964).

(7) Creagh, D. C. & McAuley, W.J.; "International Tables for Crystallography", Vol C, (A.J.C. Wilson, ed.), Kluwer Academic Publishers, Boston, Table 4.2.6.8, pages 219-222 (1992).

(8) Creagh, D. C. & Hubbell, J.H..; "International Tables for Crystallography", Vol C, (A.J.C. Wilson, ed.), Kluwer Academic Publishers, Boston, Table 4.2.4.3, pages 200-206 (1992).

(9) CrystalStructure 4.1: Crystal Structure Analysis Package, Rigaku Corporation (2000-2014). Tokyo 196-8666, Japan.

(10) SHELX2013: Sheldrick, G.M. (2013). University of Gottingen, Germany.

EXPERIMENTAL DETAILS

A. Crystal Data

Empirical Formula	$C_{14}H_5F_5IN_3$
Formula Weight	437.11
Crystal Color, Habit	colorless, prism
Crystal Dimensions	0.200 × 0.050 × 0.050 mm
Crystal System	monoclinic
Lattice Type	Primitive
Lattice Parameters	a = 25.720(2) Å<br>b = 7.5763(7) Å<br>c = 7.2135(7) Å<br>β = 92.727(3)º<br>V = 1404.1(2) $Å^3$
Space Group	P21/c (#14)
Z value	4
D_{calc}	2.068 g/cm^3
F_{000}	832.00
μ(Mo-Kα)	23.398 cm^{-1}

B. Intensity Measurements

Diffractometer	XtaLAB P100
Radiation	Mo-Kα (λ = 0.71075 Å)
Voltage, Current	45 kV, 66 mA
Temperature	−100 ºC
Detector Aperture	83.8 × 33.5 mm
Pixel Size	0.172 mm
$2\theta_{max}$	50.7º
No. of Reflections Measured	Total: 30582<br>Unique: 2577 (R_{int} = 0.0473)
Corrections	Lorentz-polarization<br>Absorption<br>(trans. factors: 0.705 – 0.890)

C. Structure Solution and Refinement

Structure Solution	Patterson Methods (DIRDIF99 PATTY)
Refinement	Full-matrix least-squares on F^2
Function Minimized	Σ w $(Fo^2 - Fc^2)^2$
Least Squares Weights	w = 1/ $[\sigma^2(Fo^2) + (0.0281\times P)^2 + 0.1920 \times P]$ where P = $(Max(Fo^2,0) + 2Fc)/3$
$2\theta_{max}$ cutoff	50.7°
Anomalous Dispersion	All non-hydrogen atoms
No. Observations (All reflections)	2577
No. Variables	208
Reflection/Parameter Ratio	12.39
Residuals: R1 (I>2.00σ(I))	0.0170
Residuals: R (All reflections)	0.0182
Residuals: wR2 (All reflections)	0.0495
Goodness of Fit Indicator	1.084
Max Shift/Error in Final Cycle	0.003
Maximum peak in Final Diff. Map	0.51 $e^-/Å^3$
Minimum peak in Final Diff. Map	−0.47 $e^-/Å^3$

4-(3,5-Di-*tert*-butylphenyl)-5-iodo-1-(perfluorophenyl)-1*H*-1,2,3-triazole (7)

Data Collection

A colorless prism crystal of $C_{22}H_{21}F_5IN_3$ having approximate dimensions of 0.170 × 0.100 × 0.090 mm was mounted in a loop. All measurements were made on a Rigaku XtaLAB P200 diffractometer using multi-layer mirror monochromated Mo-Kα radiation.

The crystal-to-detector distance was 45.05 mm.

Cell constants and an orientation matrix for data collection corresponded to a C-centered monoclinic cell with dimensions:

a = 40.169(6) Å
b = 11.3940(13) Å   β = 95.318(3)°
c = 9.9435(12) Å
V = 4531.4(10) $Å^3$

For Z = 8 and F.W. = 549.32, the calculated density is 1.610 g/cm^3. Based on the reflection conditions of:

hkl: h+k = 2n
h0l: l = 2n

packing considerations, a statistical analysis of intensity distribution, and the successful solution and refinement of the structure, the space group was determined to be:

C2/c (#15)

The data were collected at a temperature of −100 ± 1°C to a maximum 2θ value of 50.7°. A total of 2160 oscillation images were collected. A sweep of data was done using ω scans from −100.0 to 80.0° in 0.50° step, at χ = 45.0° and ϕ = 0.0°. The exposure rate was 10.0 [sec./°]. The detector

swing angle was −10.06°. A second sweep was performed using ω scans from −100.0 to 80.0° in 0.50° step, at χ = 45.0° and ϕ = 90.0°. The exposure rate was 10.0 [sec./ °]. The detector swing angle was -10.06°. Another sweep was performed using ω scans from −100.0 to 80.0° in 0.50° step, at χ = 45.0° and ϕ = 180.0°. The exposure rate was 10.0 [sec./°]. The detector swing angle was −10.06°. Another sweep was performed using ω scans from -100.0 to 80.0° in 0.50° step, at χ =45.0° and ϕ = 120.0°. The exposure rate was 10.0 [sec./°] The detector swing angle was −10.06°. Another sweep was performed using ω scans from −100.0 to 80.0° in 0.50° step, at χ =45.0° and ϕ = 240.0°. The exposure rate was 10.0 [sec./°]. The detector swing angle was -10.06°. Another sweep was performed using ω scans from −100.0 to 80.0° in 0.50° step, at χ =45.0° and ϕ = 270.0°. The exposure rate was 10.0 [sec./ °]. The detector swing angle was −10.06°. The crystal-to-detector distance was 45.05 mm. Readout was performed in the 0.172 mm pixel mode.

Data Reduction

Of the 53454 reflections were collected, where 4121 were unique (R_{int} = 0.0569); equivalent reflections were merged. Data were collected and processed using CrystalClear (Rigaku).[1]

The linear absorption coefficient, μ, for Mo-Kα radiation is 14.680 cm^{-1}. An empirical absorption correction was applied which resulted in transmission factors ranging from 0.687 to 0.876. The data were corrected for Lorentz and polarization effects.

Structure Solution and Refinement

The structure was solved by heavy-atom Patterson methods[2] and expanded using Fourier techniques. The non-hydrogen atoms were refined anisotropically. Hydrogen atoms were refined using the riding model. The final cycle of full-matrix least-squares refinement[3] on F^2 was based on 4121 observed reflections and 286 variable parameters and converged (largest parameter shift was 0.00 times its esd) with unweighted and weighted agreement factors of:

$$R1 = \Sigma \, ||Fo| - |Fc|| \, / \, \Sigma \, |Fo| = 0.0372$$

$$wR2 = [\Sigma \, (w (Fo^2 - Fc^2)^2) / \Sigma \, w(Fo^2)^2]^{1/2} = 0.1150$$

The goodness of fit[4] was 1.07. Unit weights were used. The maximum and minimum peaks on the final difference Fourier map corresponded to 1.26 and −0.35 $e^-/Å^3$, respectively.

Neutral atom scattering factors were taken from International Tables for Crystallography (IT), Vol. C, Table 6.1.1.4.[5] Anomalous dispersion effects were included in Fcalc;[6] the values for Δf' and Δf" were those of Creagh and McAuley.[7] The values for the mass attenuation coefficients are those of Creagh and Hubbell.[8] All calculations were performed using the CrystalStructure[9] crystallographic software package except for refinement, which was performed using SHELXL2013.[10]

References

(1) CrystalClear: Data Collection and Processing Software, Rigaku Corporation (1998-2014). Tokyo 196-8666, Japan.

(2) PATTY: Beurskens, P.T., Admiraal, G., Behm, H., Beurskens, G., Smits, J.M.M. and Smykalla, C. (1991). Z. f. Kristallogr. Suppl.4, p.99.

(3) Least Squares function minimized: (SHELXL2013)

$$\Sigma w(F_o^2-F_c^2)^2$$ where w = Least Squares weights.

(4) Goodness of fit is defined as:

$$[\Sigma w(F_o^2-F_c^2)^2/(N_o-N_v)]^{1/2}$$

where: N_o = number of observations
N_v = number of variables

(5) International Tables for Crystallography, Vol.C (1992). Ed. A.J.C. Wilson, Kluwer Academic Publishers, Dordrecht, Netherlands, Table 6.1.1.4, pp. 572.

(6) Ibers, J. A. & Hamilton, W. C.; Acta Crystallogr., 17, 781 (1964).

(7) Creagh, D. C. & McAuley, W.J .; "International Tables for Crystallography", Vol C, (A.J.C. Wilson, ed.), Kluwer Academic Publishers, Boston, Table 4.2.6.8, pages 219-222 (1992).

(8) Creagh, D. C. & Hubbell, J.H..; "International Tables for Crystallography", Vol C, (A.J.C. Wilson, ed.), Kluwer Academic Publishers, Boston, Table 4.2.4.3, pages 200-206 (1992).

(9) CrystalStructure 4.1: Crystal Structure Analysis Package, Rigaku Corporation (2000-2014). Tokyo 196-8666, Japan.

(10) SHELXL2013: Sheldrick, G. M. (2008). Acta Cryst. A64, 112-122.

EXPERIMENTAL DETAILS

A. Crystal Data

Empirical Formula	$C_{22}H_{21}F_5IN_3$
Formula Weight	549.32
Crystal Color, Habit	colorless, prism
Crystal Dimensions	0.170 × 0.100 × 0.090 mm
Crystal System	monoclinic
Lattice Type	C-centered
Lattice Parameters	a = 40.169(6) Å<br>b = 11.3940(13) Å<br>c = 9.9435(12) Å<br>β = 95.318(3)º<br>V = 4531.4(10) Å^3
Space Group	C2/c (#15)
Z value	8
D_{calc}	1.610 g/ cm^3
F_{000}	2176.00
μ(Mo-Kα)	14.680 cm^{-1}

B. Intensity Measurements

Diffractometer	XtaLAB P200
Radiation	MoKα (λ = 0.71075 Å)<br>multi-layer mirror monochromated
Voltage, Current	45kV, 66mA
Temperature	− 100.0 ºC
Detector Aperture	83.8 x 70.0 mm
Data Images	2160 exposures
ω oscillation Range (χ=45.0, ϕ = 0.0)	−100.0 – 80.0
Exposure Rate	10.0 sec./º
Detector Swing Angle	−10.06º
ω oscillation Range (χ=45.0, ϕ=90.0)	−100.0 – 80.0
Exposure Rate	10.0 sec./º
Detector Swing Angle	−10.06º
ω oscillation Range (χ=45.0, ϕ=180.0)	−100.0 – 80.0
Exposure Rate	10.0 sec./ º
Detector Swing Angle	−10.06º
ω oscillation Range (χ=45.0, ϕ=120.0)	−100.0 – 80.0
Exposure Rate	10.0 sec./ º
Detector Swing Angle	−10.06º
ω oscillation Range (χ=45.0, ϕ =240.0)	−100.0 – 80.0

Exposure Rate	10.0 sec./ º
Detector Swing Angle	−10.06º
ω oscillation Range (χ=45.0, φ =270.0)	−100.0 – 80.0
Exposure Rate	10.0 sec./ º
Detector Swing Angle	−10.06º
Detector Position	45.05 mm
Pixel Size	0.172 mm
$2\theta_{max}$	50.7º
No. of Reflections Measured	Total: 53454<br>Unique: 4121 (R_{int} = 0.0569)
Corrections	Lorentz-polarization<br>Absorption<br>(trans. factors: 0.687 – 0.876)

C. Structure Solution and Refinement

Structure Solution	Patterson Methods (DIRDIF99 PATTY)
Refinement	Full-matrix least-squares on F^2
Function Minimized	Σ w $(Fo^2 - Fc^2)^2$
Least Squares Weights	$w = 1/ [\sigma^2(Fo^2) + (0.0281\times P)^2 + 0.1920 \times P]$ where $P = (Max(Fo^2,0) + 2Fc)/3$
$2\theta_{max}$ cutoff	50.7º
Anomalous Dispersion	All non-hydrogen atoms
No. Observations (All reflections)	4121
No. Variables	286
Reflection/Parameter Ratio	14.41
Residuals: R1 ($I>2.00\sigma(I)$)	0.0372
Residuals: R (All reflections)	0.0403
Residuals: wR2 (All reflections)	0.1150
Goodness of Fit Indicator	1.069
Max Shift/Error in Final Cycle	0.002
Maximum peak in Final Diff. Map	1.26 e^-/ $Å^3$
Minimum peak in Final Diff. Map	−0.35 e^-/$Å^3$

3-(4-(4-(3,5-Di-tert-butylphenyl)-5-iodo-1H-1,2,3-triazol-1-yl)-2,3,5,6-tetrafluorophenoxy)pyridine (9a)

Data Collection

A colorless prism crystal of $C_{27}H_{25}F_4IN_4O$ having approximate dimensions of 0.150 × 0.100 × 0.030 mm was mounted in a loop. All measurements were made on a Rigaku XtaLAB P200 diffractometer using multi-layer mirror monochromated Mo-Kα radiation.

Cell constants and an orientation matrix for data collection corresponded to a primitive triclinic cell with dimensions:

a = 10.646(6) Å   α = 93.081(7)°
b = 11.018(6) Å   β = 96.436(12)°
c = 12.134(5) Å   γ = 113.187(11)°
V = 1292.6(12) $Å^3$

For Z = 2 and F.W. = 624.42, the calculated density is 1.604 g/cm^3. Based on a statistical analysis of intensity distribution, and the successful solution and refinement of the structure, the space group was determined to be:

P-1 (#2)

The data were collected at a temperature of −180 ± 1°C to a maximum 2θ value of 50.7°.

Data Reduction

Of the 17633 reflections were collected, where 4696 were unique (R_{int} = 0.0894); equivalent reflections were merged. Data were collected and processed using CrystalClear (Rigaku).[1]

The linear absorption coefficient, μ, for Mo-Kα radiation is 12.959 cm^{-1}. An empirical absorption correction was applied which resulted in transmission factors ranging from 0.460 to 0.962. The data were corrected for Lorentz and polarization effects.

Structure Solution and Refinement

The structure was solved by direct methods[2] and expanded using Fourier techniques. The non-hydrogen atoms were refined anisotropically. Hydrogen atoms were refined using the riding model. The final cycle of full-matrix least-squares refinement[3] on F^2 was based on 4696 observed reflections and 340 variable parameters and converged (largest parameter shift was 0.00 times its esd) with unweighted and weighted agreement factors of:

$$R1 = \Sigma ||Fo| - |Fc|| / \Sigma |Fo| = 0.0381$$

$$wR2 = [\Sigma (w (Fo^2 - Fc^2)^2)/ \Sigma w(Fo^2)^2]^{1/2} = 0.0983$$

The goodness of fit[4] was 0.92. Unit weights were used. Plots of Σ w(|Fo| − |Fc|)^2 versus |Fo|, reflection order in data collection, sin θ/λ and various classes of indices showed no unusual trends. The maximum and minimum peaks on the final difference Fourier map corresponded to 1.93 and −0.69 $e^-/Å^3$, respectively.

Neutral atom scattering factors were taken from International Tables for Crystallography (IT), Vol. C, Table 6.1.1.4.[5] Anomalous dispersion effects were included in Fcalc;[6] the values for Δf' and Δf" were those of Creagh and McAuley.[7] The values for the mass attenuation coefficients are those of Creagh and Hubbell.[8] All calculations were performed using the CrystalStructure[9] crystallographic software package except for refinement, which was performed using SHELXL-2013.[10]

References

(1) CrystalClear: Data Collection and Processing Software, Rigaku Corporation (1998-2014). Tokyo 196-8666, Japan.

(2) SIR2011: Burla, M. C., Caliandro, R., Camalli, M., Carrozzini, B., Cascarano, G. L., Giacovazzo, C., Mallamo, M., Mazzone, A., Polidori, G. and Spagna, R. (2012). J. Appl. Cryst. 45, 357-361.

(3) Least Squares function minimized: (SHELXL2013)

$\Sigma w(F_o^2-F_c^2)^2$ where w = Least Squares weights.

(4) Goodness of fit is defined as:

$$[\Sigma w(F_o^2-F_c^2)^2/(N_o-N_v)]^{1/2}$$

where: N_o = number of observations

N_v = number of variables

(5) International Tables for Crystallography, Vol.C (1992). Ed. A.J.C. Wilson, Kluwer Academic Publishers, Dordrecht, Netherlands, Table 6.1.1.4, pp. 572.

(6) Ibers, J. A. & Hamilton, W. C.; Acta Crystallogr., 17, 781 (1964).

(7) Creagh, D. C. & McAuley, W.J .; "International Tables for Crystallography", Vol C, (A.J.C. Wilson, ed.), Kluwer Academic Publishers, Boston, Table 4.2.6.8, pages 219-222 (1992).

(8) Creagh, D. C. & Hubbell, J.H..; "International Tables for Crystallography", Vol C, (A.J.C. Wilson, ed.), Kluwer Academic Publishers, Boston, Table 4.2.4.3, pages 200-206 (1992).

(9) CrystalStructure 4.1: Crystal Structure Analysis Package, Rigaku Corporation (2000-2014). Tokyo 196-8666, Japan.

(10) SHELXL2013: Sheldrick, G. M. (2008). Acta Cryst. A64, 112-122.

EXPERIMENTAL DETAILS

A. Crystal Data

Empirical Formula	$C_{27}H_{25}F_4IN_4O$
Formula Weight	624.42
Crystal Color, Habit	colorless, prism
Crystal Dimensions	0.150 × 0.100 × 0.030 mm
Crystal System	triclinic
Lattice Type	Primitive
Lattice Parameters	a = 10.646(6) Å<br>b = 11.018(6) Å<br>c = 12.134(5) Å<br>α = 93.081(7)º<br>β= 96.436(12)º<br>γ = 113.187(11)º<br>V = 1292.6(12) Å^3
Space Group	P-1 (#2)
Z value	2
D_{calc}	1.604 g/cm^3
F_{000}	624.00
μ(Mo-Kα)	12.959 cm^{-1}

B. Intensity Measurements

Diffractometer	XtaLAB P200
Radiation	MoKα (λ = 0.71075 Å)<br>multi-layer mirror monochromated
Voltage, Current	45kV, 66mA
Temperature	−180.0º C
Detector Aperture	83.8 × 70.0 mm
Data Images	1322 exposures
ω oscillation Range (χ=60.0, φ=−180.0)	−80.0 – 100.0º
Exposure Rate	6.0 sec./º
Detector Swing Angle	10.11º
ω oscillation Range (χ=60.0, φ=0.0)	−62.0 – 100.0º
Exposure Rate	6.0 sec./º
Detector Swing Angle	10.11º
ω oscillation Range (χ=60.0, φ=−90.0)	−62.0 – 100.0º
Exposure Rate	6.0 sec./º
Detector Swing Angle	10.11º
ω oscillation Range (χ=60.0, φ=90.0)	−62.0 – 95.0º
Exposure Rate	6.0 sec./º
Detector Swing Angle	10.11º
Detector Position	48.87 mm
Pixel Size	0.172 mm
$2\theta_{max}$	50.7º
No. of Reflections Measured	Total: 17633<br>Unique: 4696 (R_{int} = 0.0894)

Corrections	Lorentz-polarization<br>Absorption<br>(trans. factors: 0.460 – 0.962)

C. Structure Solution and Refinement

Structure Solution	Direct Methods (SIR2011)
Refinement	Full-matrix least-squares on F^2
Function Minimized	Σ w $(Fo^2 - Fc^2)^2$
Least Squares Weights	w = 1/ $[\sigma^2(Fo^2) + (0.0598\times P)^2 + 0.0000 \times P]$ where P = $(Max(Fo^2,0) + 2Fc^2)/3$
$2\theta_{max}$ cutoff	50.7º
Anomalous Dispersion	All non-hydrogen atoms
No. Observations (All reflections)	4696
No. Variables	340
Reflection/Parameter Ratio	13.81
Residuals: R1 ($I>2.00\sigma(I)$)	0.0381
Residuals: R (All reflections)	0.0402
Residuals: wR2 (All reflections)	0.0983
Goodness of Fit Indicator	0.917
Max Shift/Error in Final Cycle	0.001
Maximum peak in Final Diff. Map	1.93 $e^-/Å^3$
Minimum peak in Final Diff. Map	−0.69 $e^-/Å^3$

5-iodo-4-phenyl-1-(2,3,5,6-tetrafluoro-[1,1'-biphenyl]-4-yl)-1*H*-1,2,3-triazole (**S4**)

Data Collection

A colorless prism crystal of $C_{20}H_{10}F_4IN_3$ having approximate dimensions of 0.210 × 0.100 × 0.070 mm was mounted in a loop. All measurements were made on a Rigaku XtaLAB P200 diffractometer using multi-layer mirror monochromated Mo-Kα radiation.

The crystal-to-detector distance was 45.01 mm.

Cell constants and an orientation matrix for data collection corresponded to a primitive orthorhombic cell with dimensions:

a = 7.1624(11) Å
b = 7.4352(13) Å
c = 33.215(5) Å
V = 1768.8(5) $Å^3$

For Z = 4 and F.W. = 495.22, the calculated density is 1.859 g/cm^3. Based on the reflection conditions of:

0kl: k+l = 2n
h0l: h = 2n

packing considerations, a statistical analysis of intensity distribution, and the successful solution and refinement of the structure, the space group was determined to be:

$Pna2_1$ (#33)

The data were collected at a temperature of −100 + 1 ºC to a maximum 2θ value of 50.8º. A total of 1800 oscillation images were collected. A sweep of data was done using ω scans from −100.0 to 80.0º in 0.50º step, at χ = 45.0º and ϕ = 0.0º. The exposure rate was 6.0 [sec./º]. The detector swing angle was −10.39º. A second sweep was performed using ω scans from −100.0 to 80.0º in 0.50º step, at χ=45.0º and ϕ = 90.0º. The exposure rate was 6.0 [sec./º]. The detector swing angle was

−10.39°. Another sweep was performed using ω scans from -100.0 to 80.0° in 0.50° step, at χ =45.0° and ϕ = 180.0°. The exposure rate was 6.0 [sec./°]. The detector swing angle was -10.39°. Another sweep was performed using ω scans from -100.0 to 80.0° in 0.50° step, at χ =45.0° and ϕ = 111.0°. The exposure rate was 6.0 [sec./°]. The detector swing angle was -10.39°. Another sweep was performed using ω scans from -100.0 to 80.0° in 0.50° step, at χ =45.0° and ϕ = 83.0°. The exposure rate was 6.0 [sec./°]. The detector swing angle was −10.39°. The crystal-to-detector distance was 45.01 mm. Readout was performed in the 0.086 mm pixel mode.

Data Reduction

Of the 31059 reflections were collected, where 3220 were unique (R_{int} = 0.0702); equivalent reflections were merged. Data were collected and processed using CrystalClear (Rigaku).[1]

The linear absorption coefficient, μ, for Mo-Kα radiation is 18.620 cm^{-1}. An empirical absorption correction was applied which resulted in transmission factors ranging from 0.672 to 0.878. The data were corrected for Lorentz and polarization effects.

Structure Solution and Refinement

The structure was solved by direct methods[2] and expanded using Fourier techniques. The non-hydrogen atoms were refined anisotropically. Hydrogen atoms were refined using the riding model. The final cycle of full-matrix least-squares refinement[3] on F^2 was based on 3220 observed reflections and 253 variable parameters and converged (largest parameter shift was 0.00 times its esd) with unweighted and weighted agreement factors of:

$$R1 = \Sigma ||Fo| - |Fc|| / \Sigma |Fo| = 0.0291$$

$$wR2 = [\Sigma (w (Fo^2 - Fc^2)^2)/ \Sigma w(Fo^2)^2]^{1/2} = 0.0520$$

The goodness of fit[4] was 1.01. Unit weights were used. The maximum and minimum peaks on the final difference Fourier map corresponded to 0.63 and −0.47 $e^-/Å^3$, respectively. The final Flack parameter[5] was −0.023(15), indicating that the present absolute structure is correct.[6]

Neutral atom scattering factors were taken from International Tables for Crystallography (IT), Vol. C, Table 6.1.1.4.[7] Anomalous dispersion effects were included in Fcalc;[8] the values for Δf' and Δf''

were those of Creagh and McAuley.[9] The values for the mass attenuation coefficients are those of Creagh and Hubbel.[10] All calculations were performed using the CrystalStructure[11] crystallographic software package except for refinement, which was performed using SHELXL2013.[12]

References

(1) CrystalClear: Data Collection and Processing Software, Rigaku Corporation (1998-2014). Tokyo 196-8666, Japan.

(2) SIR2011: Burla, M. C., Caliandro, R., Camalli, M., Carrozzini, B., Cascarano, G. L., Giacovazzo, C., Mallamo, M., Mazzone, A., Polidori, G. and Spagna, R. (2012). J. Appl. Cryst. 45, 357-361.

(3) Least Squares function minimized: (SHELXL2013)

$\Sigma w(F_o^2 - F_c^2)^2$ where w = Least Squares weights.

(4) Goodness of fit is defined as:

$[\Sigma w(F_o^2 - F_c^2)^2/(N_o - N_v)]^{1/2}$

where: N_o = number of observations

N_v = number of variables

(5) Parsons, S. and Flack, H. (2004), Acta Cryst. A60, s61.

(6) Flack, H.D. and Bernardinelli (2000), J. Appl. Cryst. 33, 114-1148.

(7) International Tables for Crystallography, Vol.C (1992). Ed. A.J.C. Wilson, Kluwer Academic Publishers, Dordrecht, Netherlands, Table 6.1.1.4, pp. 572.

(8) Ibers, J. A. & Hamilton, W. C.; Acta Crystallogr., 17, 781 (1964).

(9) Creagh, D. C. & McAuley, W.J .; "International Tables for Crystallography", Vol C, (A.J.C. Wilson, ed.), Kluwer Academic Publishers, Boston, Table 4.2.6.8, pages 219-222 (1992).

(10) Creagh, D. C. & Hubbell, J.H..; "International Tables for Crystallography", Vol C, (A.J.C. Wilson, ed.), Kluwer Academic Publishers, Boston, Table 4.2.4.3, pages 200-206 (1992).

(11) CrystalStructure 4.1: Crystal Structure Analysis Package, Rigaku Corporation (2000-2014). Tokyo 196-8666, Japan.

(12) SHELXL2013: Sheldrick, G. M. (2008). Acta Cryst. A64, 112-122.

EXPERIMENTAL DETAILS

A. Crystal Data

Empirical Formula	$C_{20}H_{10}F_4IN_3$
Formula Weight	495.22
Crystal Color, Habit	colorless, prism
Crystal Dimensions	0.210 × 0.100 × 0.070 mm
Crystal System	orthorhombic
Lattice Type	Primitive
Lattice Parameters	a = 7.1624(11) Å<br>b = 7.4352(13) Å<br>c = 33.215(5) Å<br>V = 1768.8(5) Å^3
Space Group	$Pna2_1$ (#33)
Z value	4
D_{calc}	1.859 g/ cm^3
F_{000}	960.00
μ(Mo-Kα)	18.620 cm^{-1}

B. Intensity Measurements

Diffractometer	XtaLAB P200
Radiation	MoKα (λ = 0.71075 Å)<br>multi-layer mirror monochromated
Voltage, Current	45kV, 66mA
Temperature	−100.0 ºC
Detector Aperture	83.8 × 70.0 mm
Data Images	1800 exposures
ω oscillation Range (χ=45.0, ϕ=0.0)	−100.0 – 80.0º
Exposure Rate	6.0 sec./º
Detector Swing Angle	−10.39º
ω oscillation Range (χ=45.0, ϕ=90.0)	−100.0 – 80.0º
Exposure Rate	6.0 sec./º
Detector Swing Angle	−10.39º
ω oscillation Range (χ=45.0, ϕ=180.0)	−100.0 – 80.0º
Exposure Rate	6.0 sec./º
Detector Swing Angle	−10.39º
ω oscillation Range (χ=45.0, ϕ=111.0)	−100.0 – 80.0º
Exposure Rate	6.0 sec./º
Detector Swing Angle	−10.39º
ω oscillation Range (χ=45.0, ϕ=83.0)	−100.0 – 80.0º
Exposure Rate	6.0 sec./º
Detector Swing Angle	−10.39º
Detector Position	45.01 mm
Pixel Size	0.086 mm

$2\theta_{max}$	50.8º
No. of Reflections Measured	Total: 31059<br>Unique: 3220 (R_{int} = 0.0702)<br>Parsons quotients (Flack × parameter): 1334
Corrections	Lorentz-polarization<br>Absorption<br>(trans. factors: 0.672 – 0.878)

C. Structure Solution and Refinement

Structure Solution	Direct Methods (SIR2011)
Refinement	Full-matrix least-squares on F^2
Function Minimized	Σ w $(Fo^2 - Fc^2)^2$
Least Squares Weights	$w = 1/ [\sigma^2(Fo^2) + (0.0265\times P)^2 + 0.0000 \times P]$ where $P = (Max(Fo^2,0) + 2Fc^2)/3$
$2\theta_{max}$ cutoff	50.8º
Anomalous Dispersion	All non-hydrogen atoms
No. Observations (All reflections)	3220
No. Variables	253
Reflection/Parameter Ratio	12.73
Residuals: R1 (I>2.00σ(I))	0.0219
Residuals: R (All reflections)	0.0234
Residuals: wR2 (All reflections)	0.0520
Goodness of Fit Indicator	1.011
Flack parameter (Parsons' quotients = 1334)	−0.023(15)
Max Shift/Error in Final Cycle	0.001
Maximum peak in Final Diff. Map	0.63 $e^-/Å^3$
Minimum peak in Final Diff. Map	−0.47 $e^-/Å^3$

S8. NMR spectra

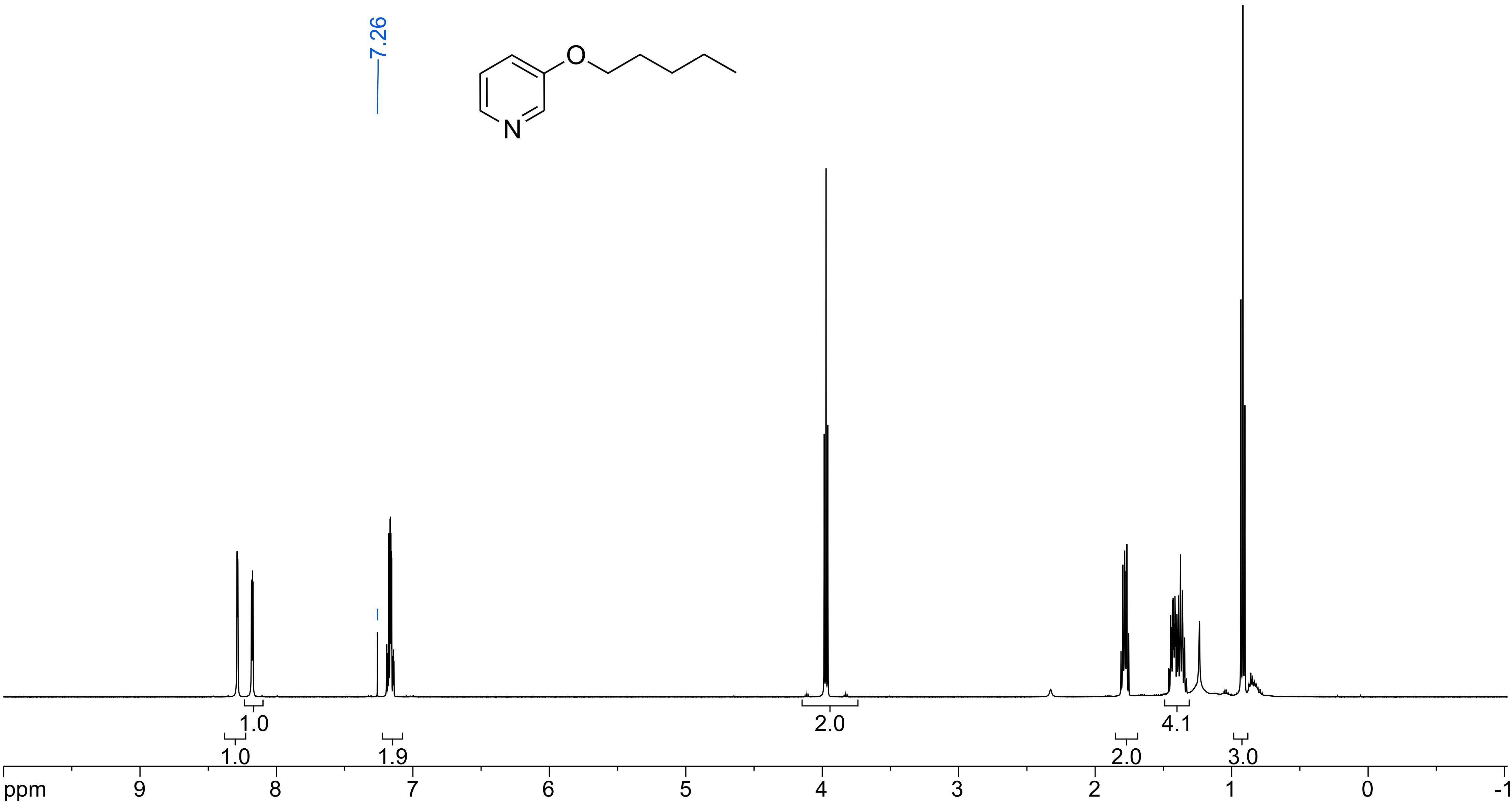

^{1}H NMR (500.1 MHz, RT, $CDCl_3$) spectrum of 3-pentyloxy-pyridine **8**

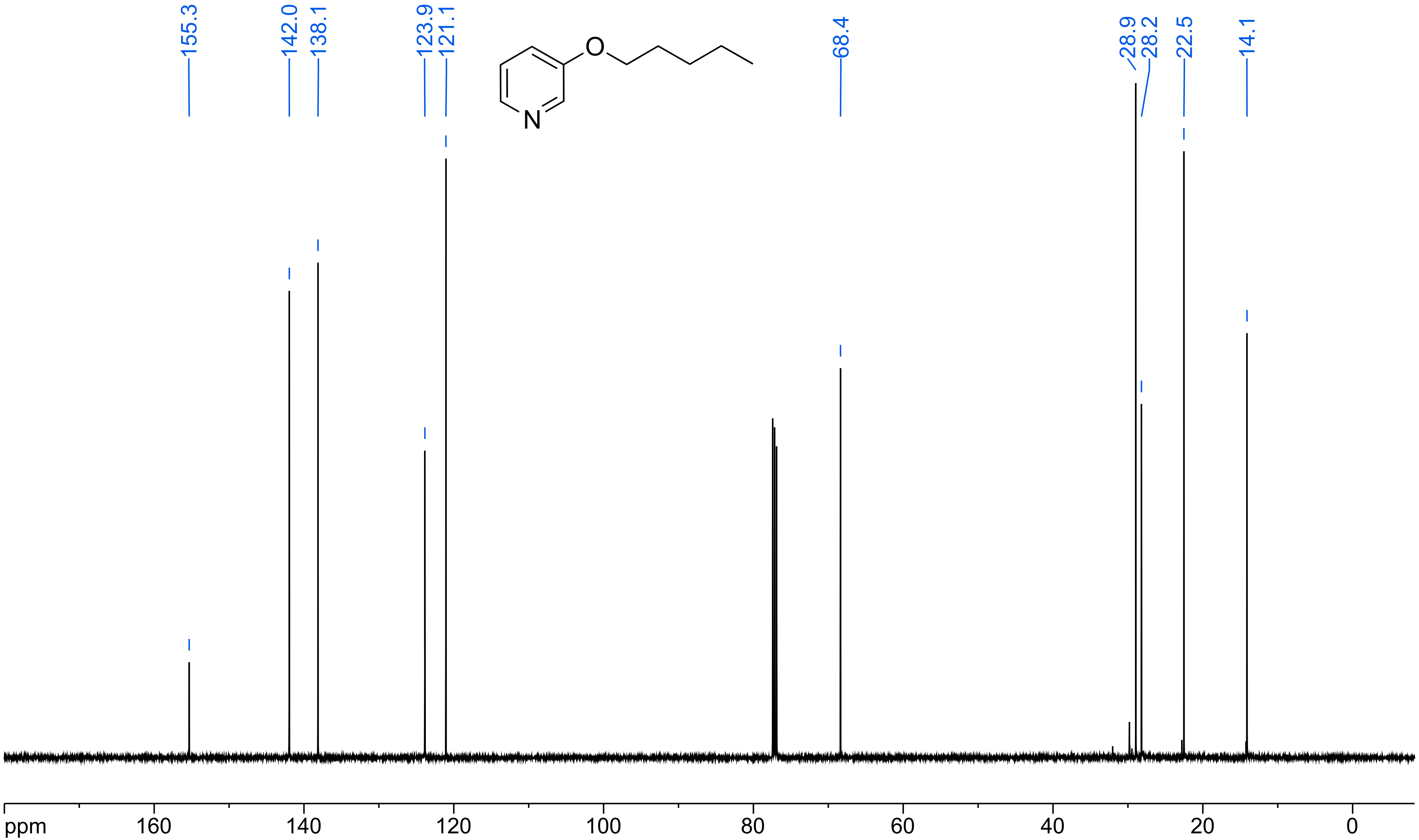

^{13}C NMR (125.7 MHz, RT, $CDCl_3$) spectrum of 3-pentyloxy-pyridine **8**

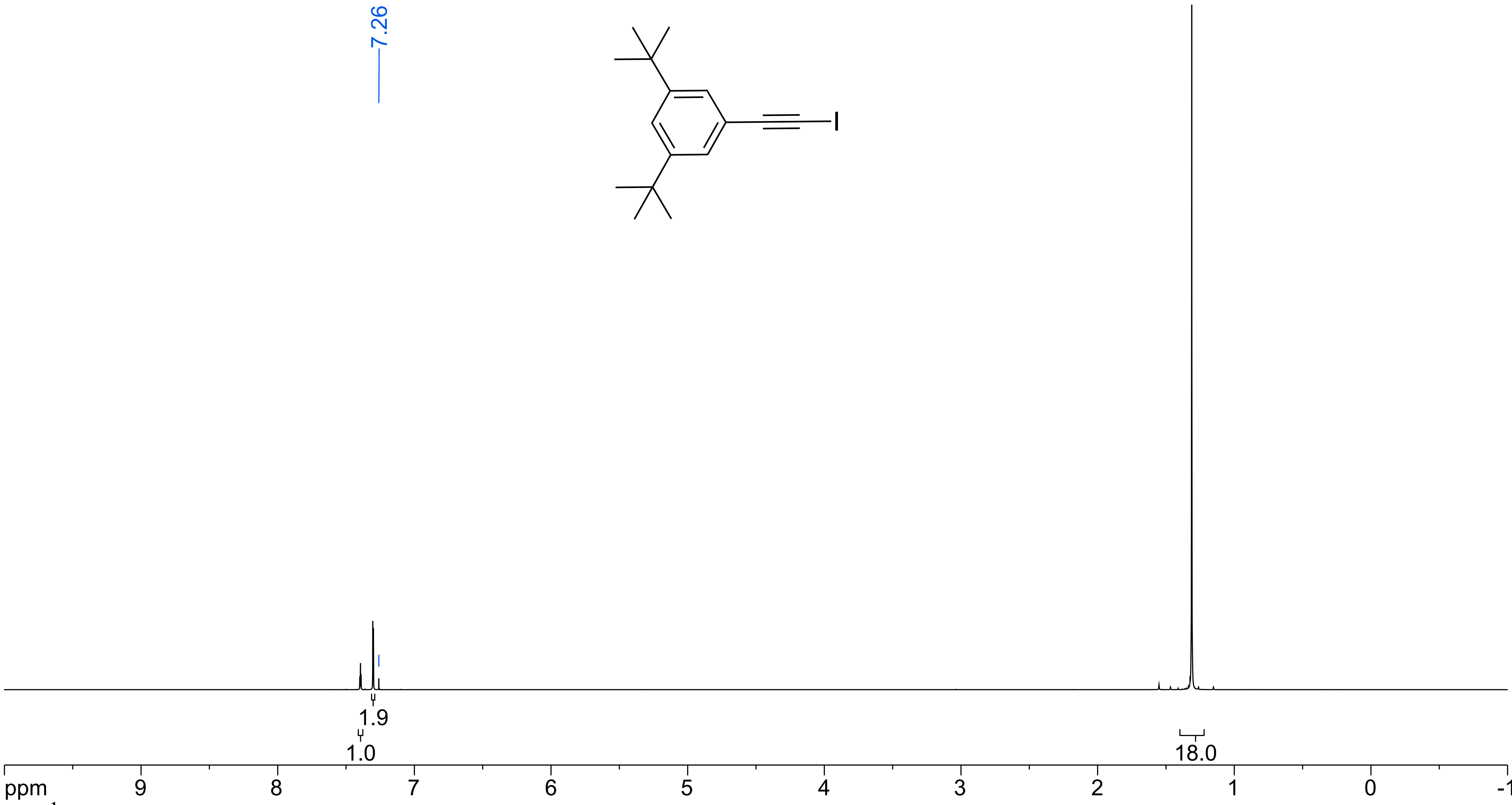

^{1}H NMR (500.1 MHz, RT, $CDCl_3$) spectrum of iodoalkyne **S1**

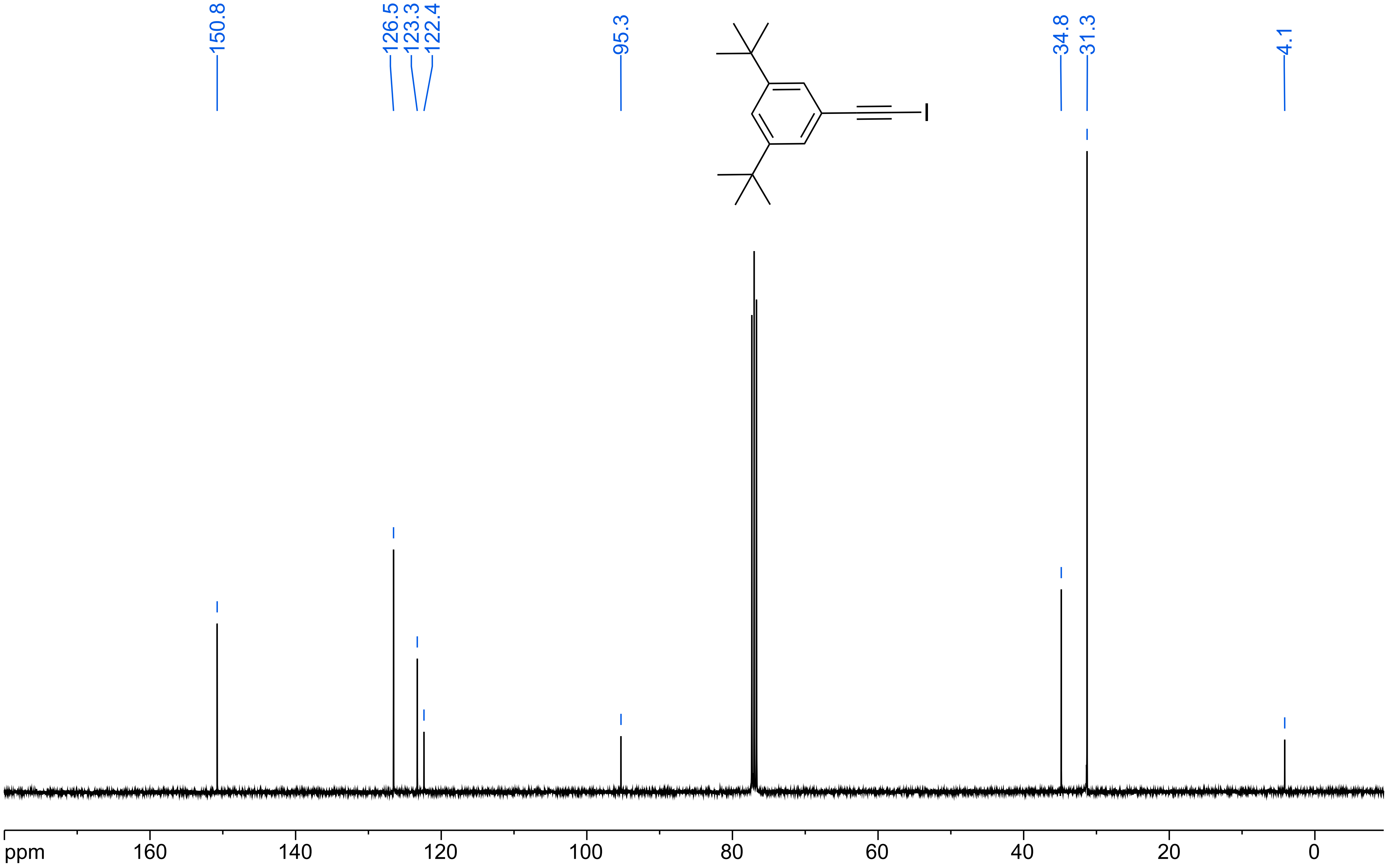

^{13}C NMR (125.7 MHz, RT, $CDCl_3$) spectrum of iodoalkyne **S1**

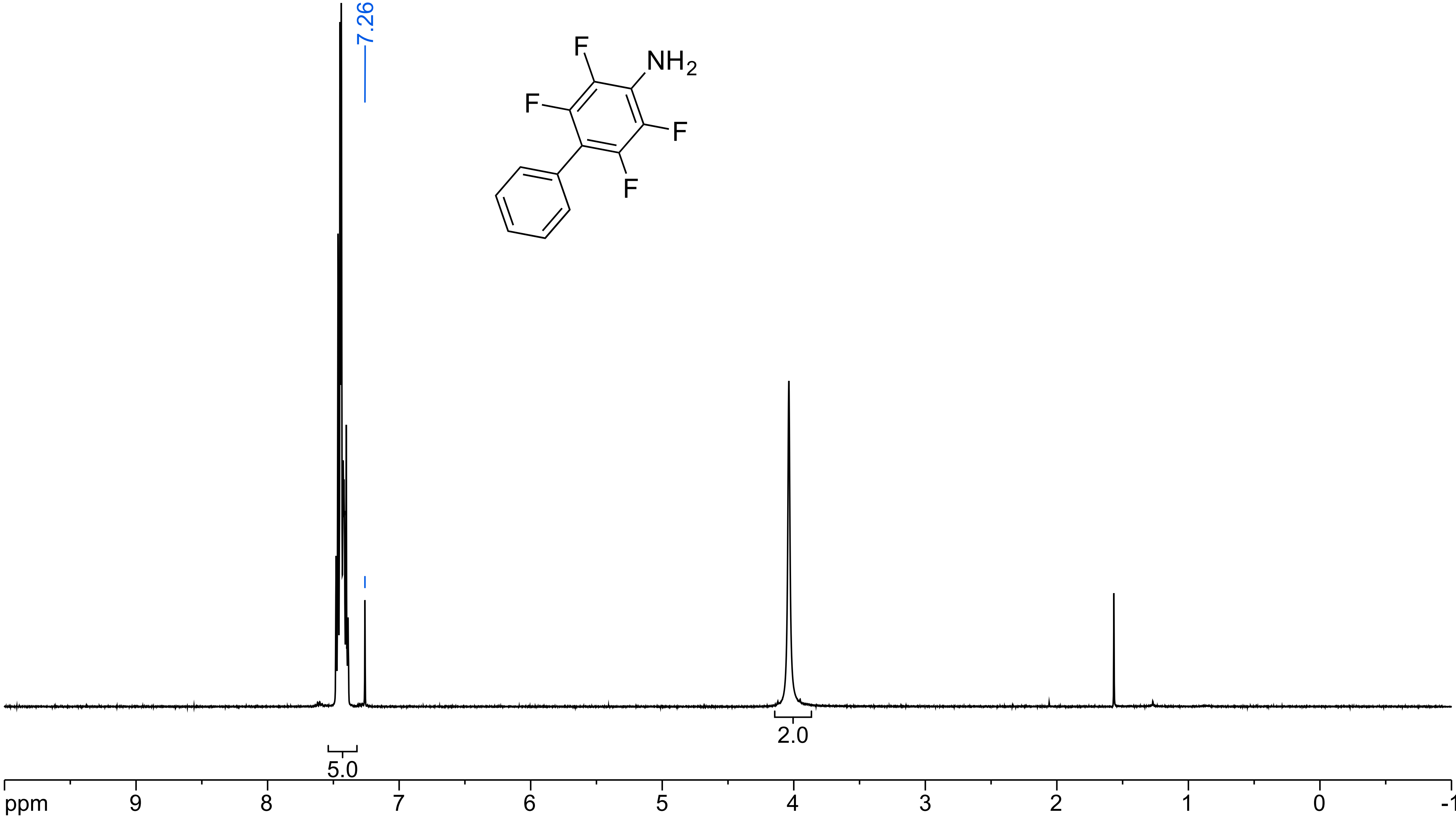

^{1}H NMR (500.1 MHz, RT, $CDCl_3$) spectrum of aniline **S2**

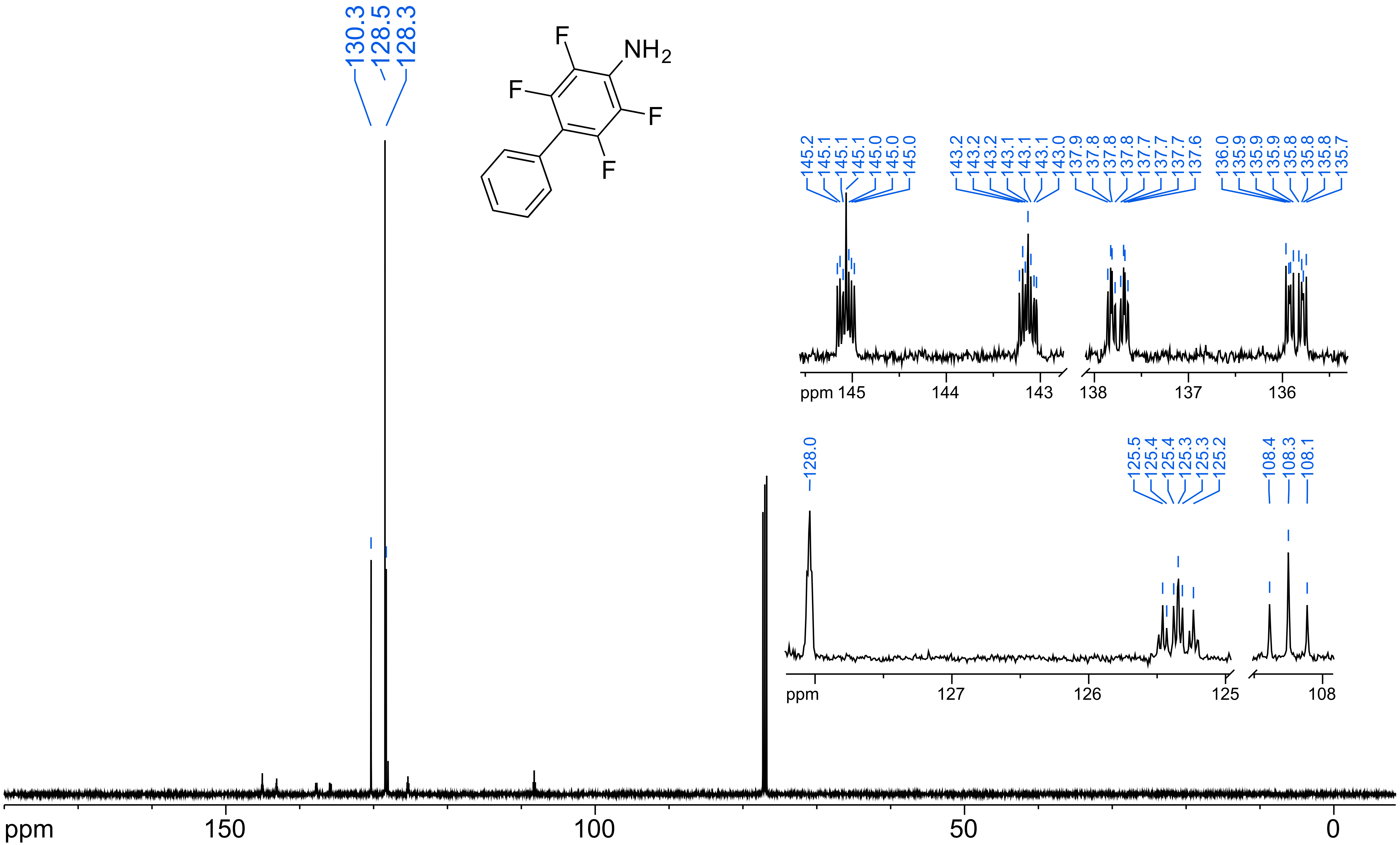

^{13}C NMR (125.7 MHz, RT, $CDCl_3$) spectrum of aniline **S2**

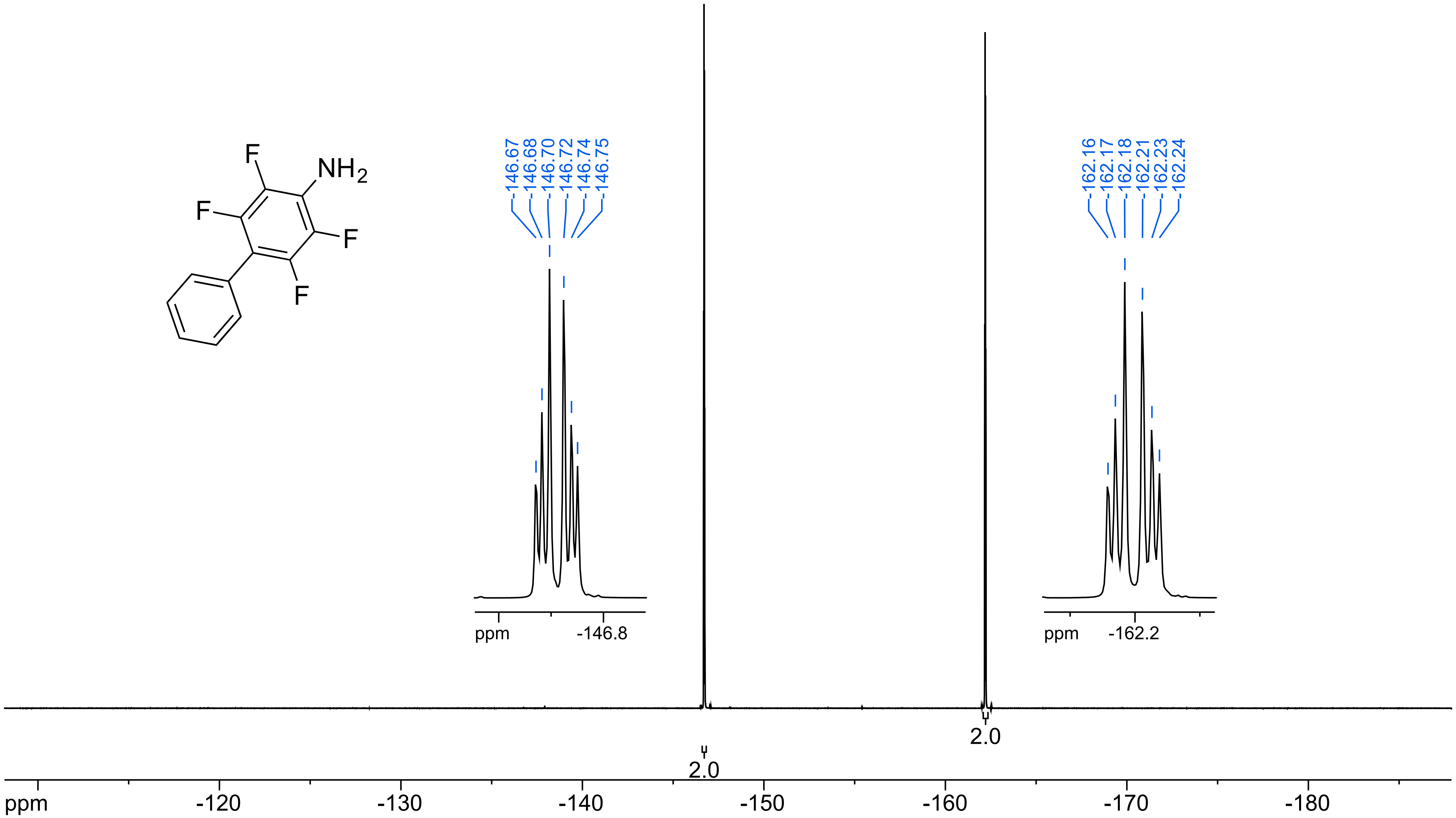

^{19}F NMR (470.5 MHz, RT, $CDCl_3$) spectrum of aniline **S2**

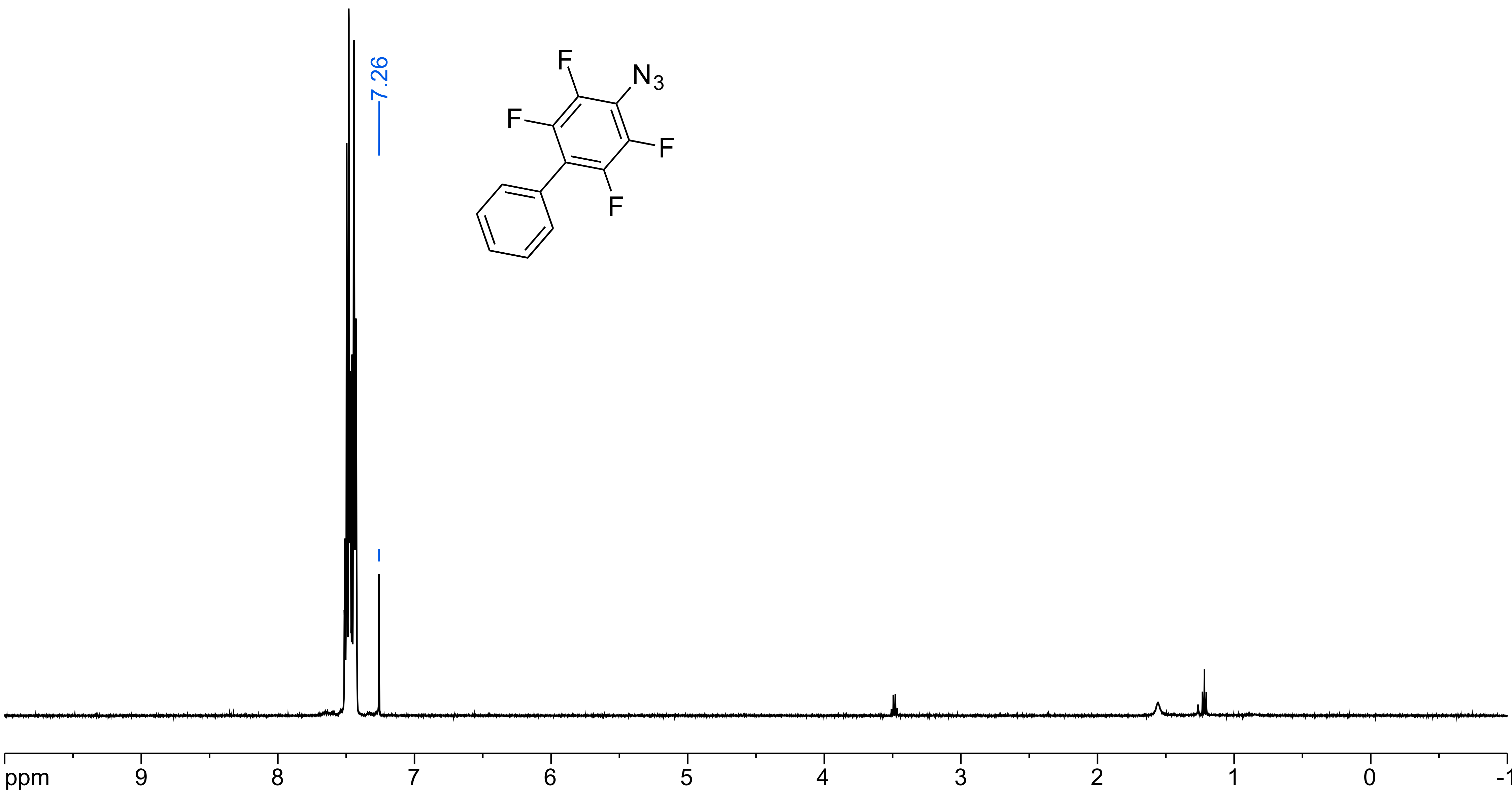

^{1}H NMR (500.1 MHz, RT, $CDCl_3$) spectrum of azide **S3**

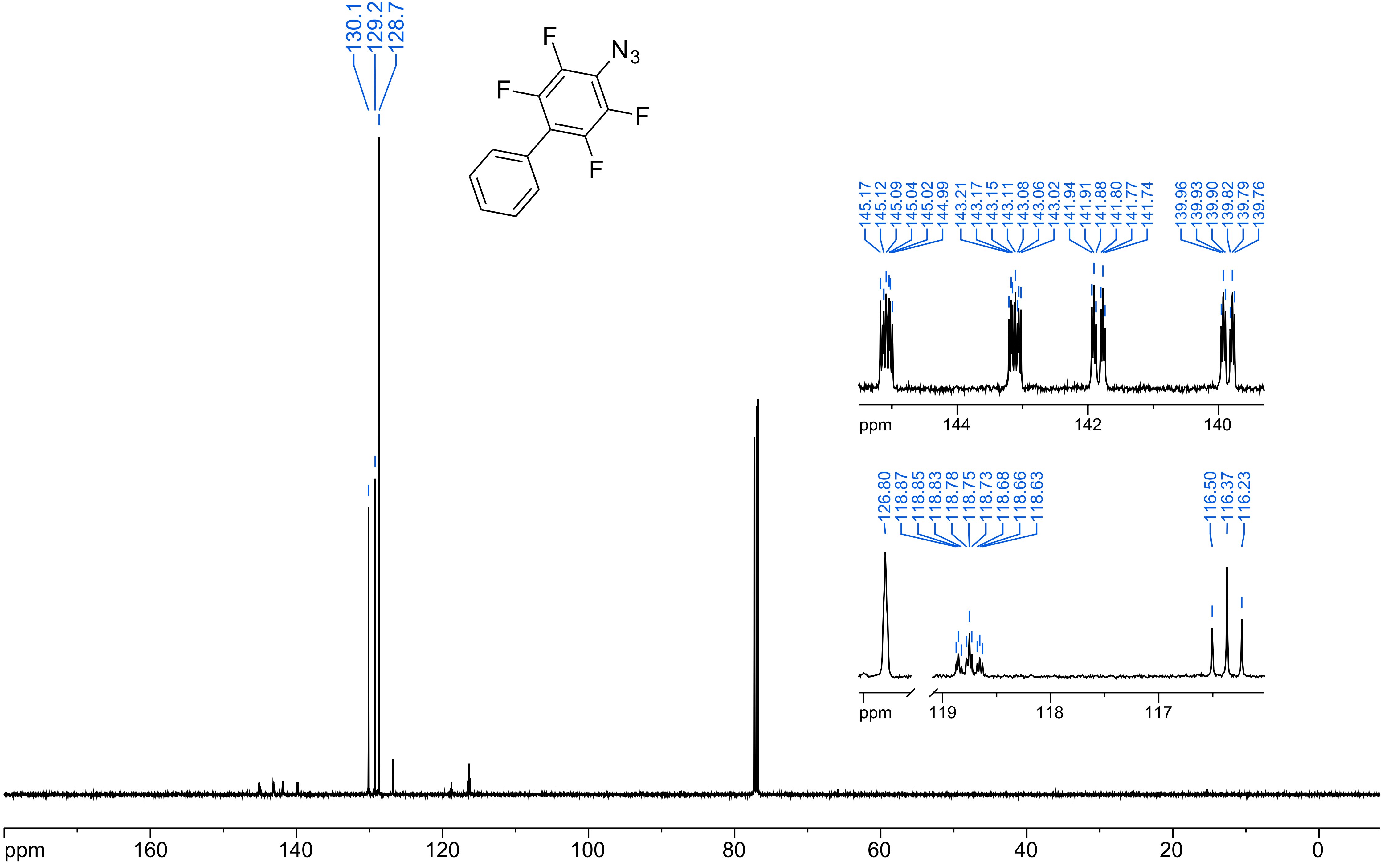

^{13}C NMR (125.7 MHz, RT, $CDCl_3$) spectrum of azide **S3**

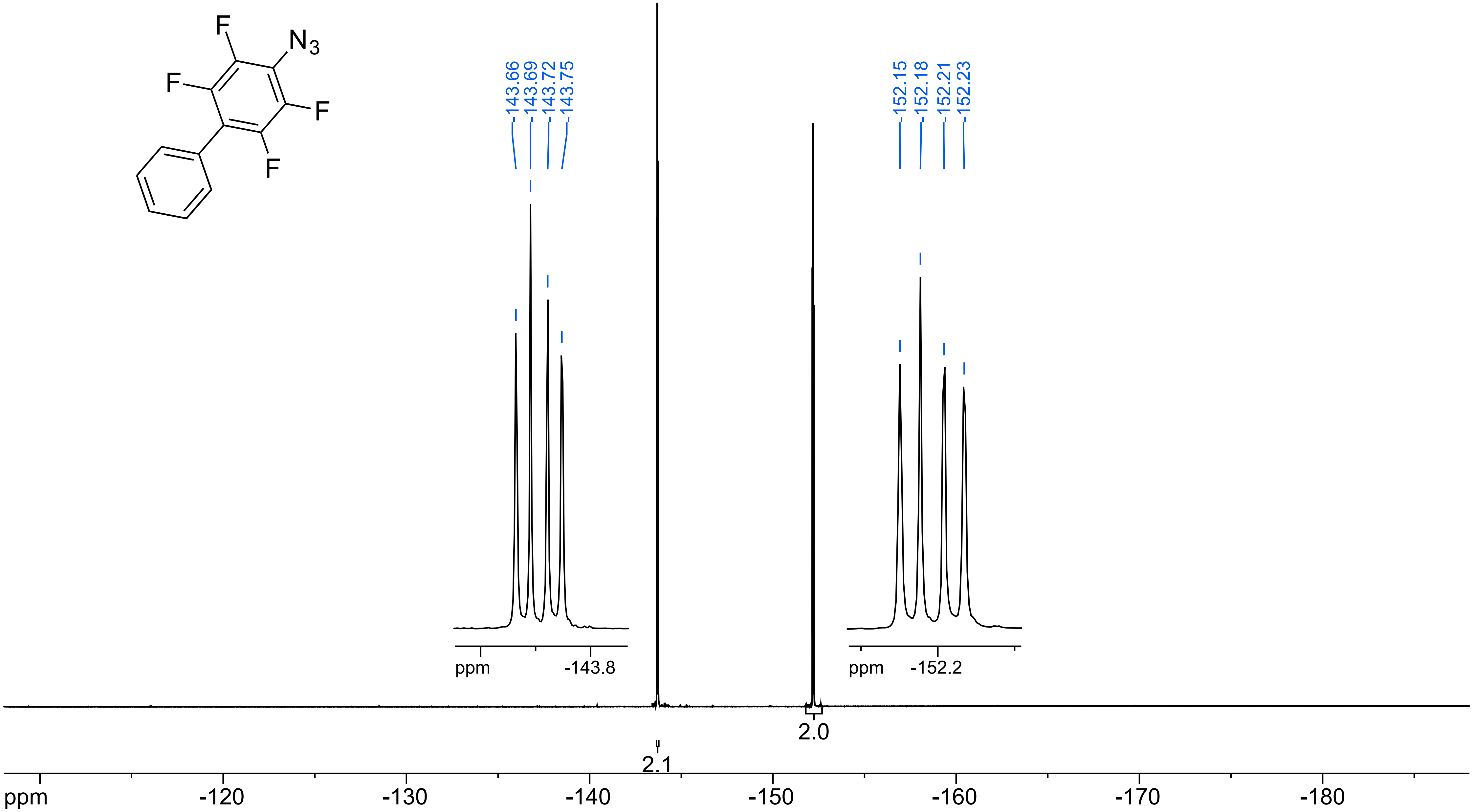

^{19}F NMR (470.5 MHz, RT, $CDCl_3$) spectrum of azide **S3**

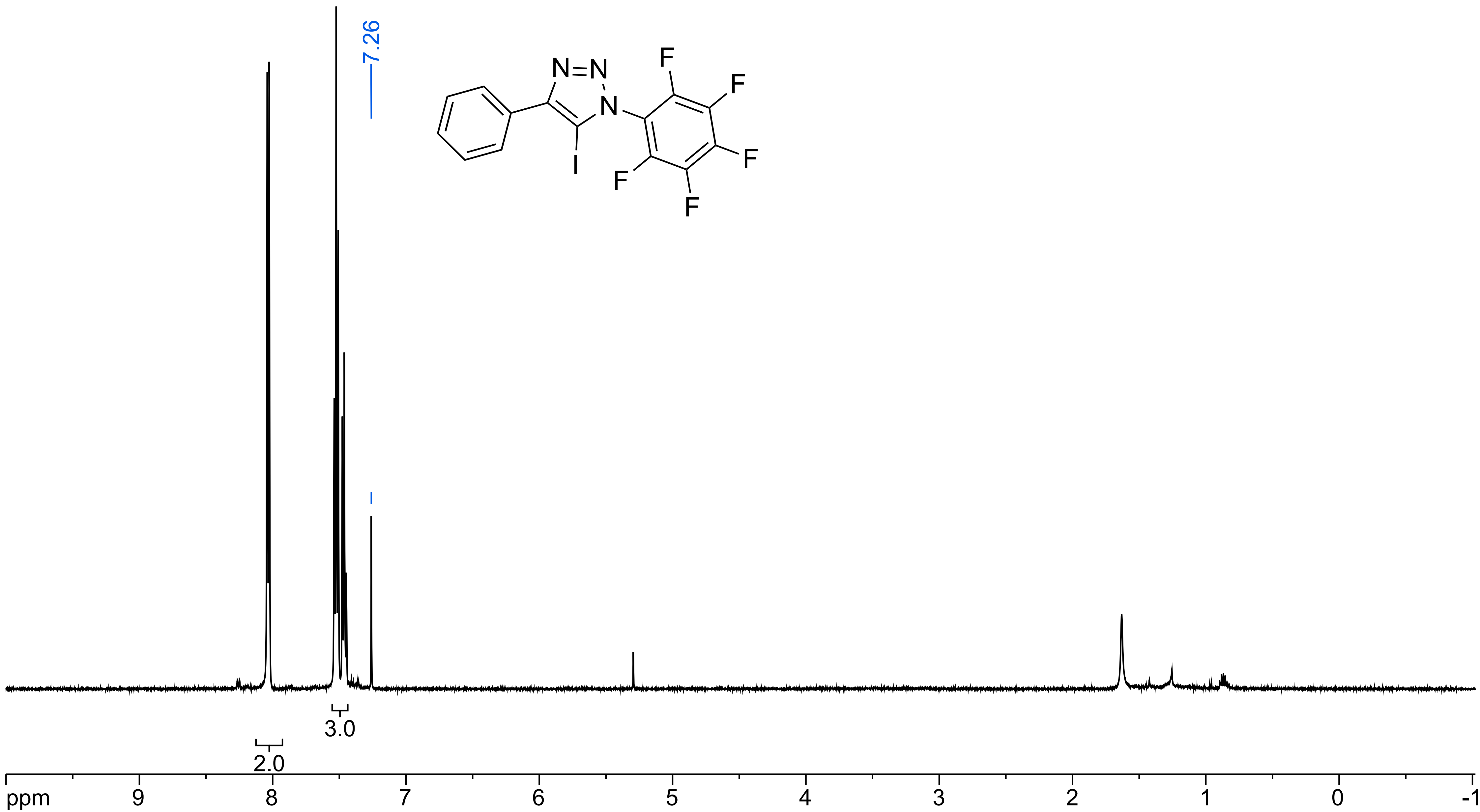

^{1}H NMR (500.1 MHz, RT, $CDCl_3$) spectrum of iodotriazole **1**

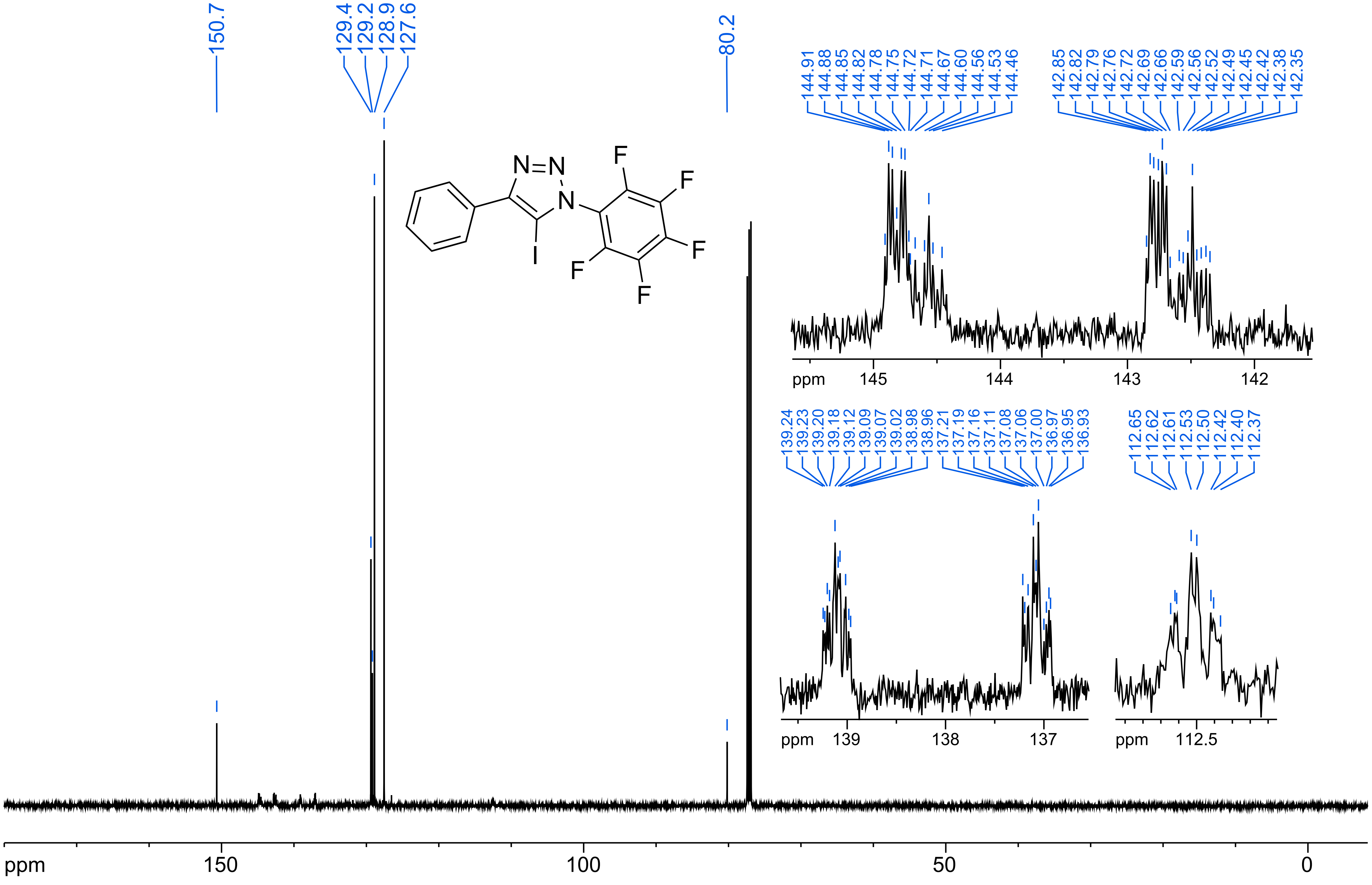

^{13}C NMR (125.7 MHz, RT, $CDCl_3$) spectrum of iodotriazole **1**

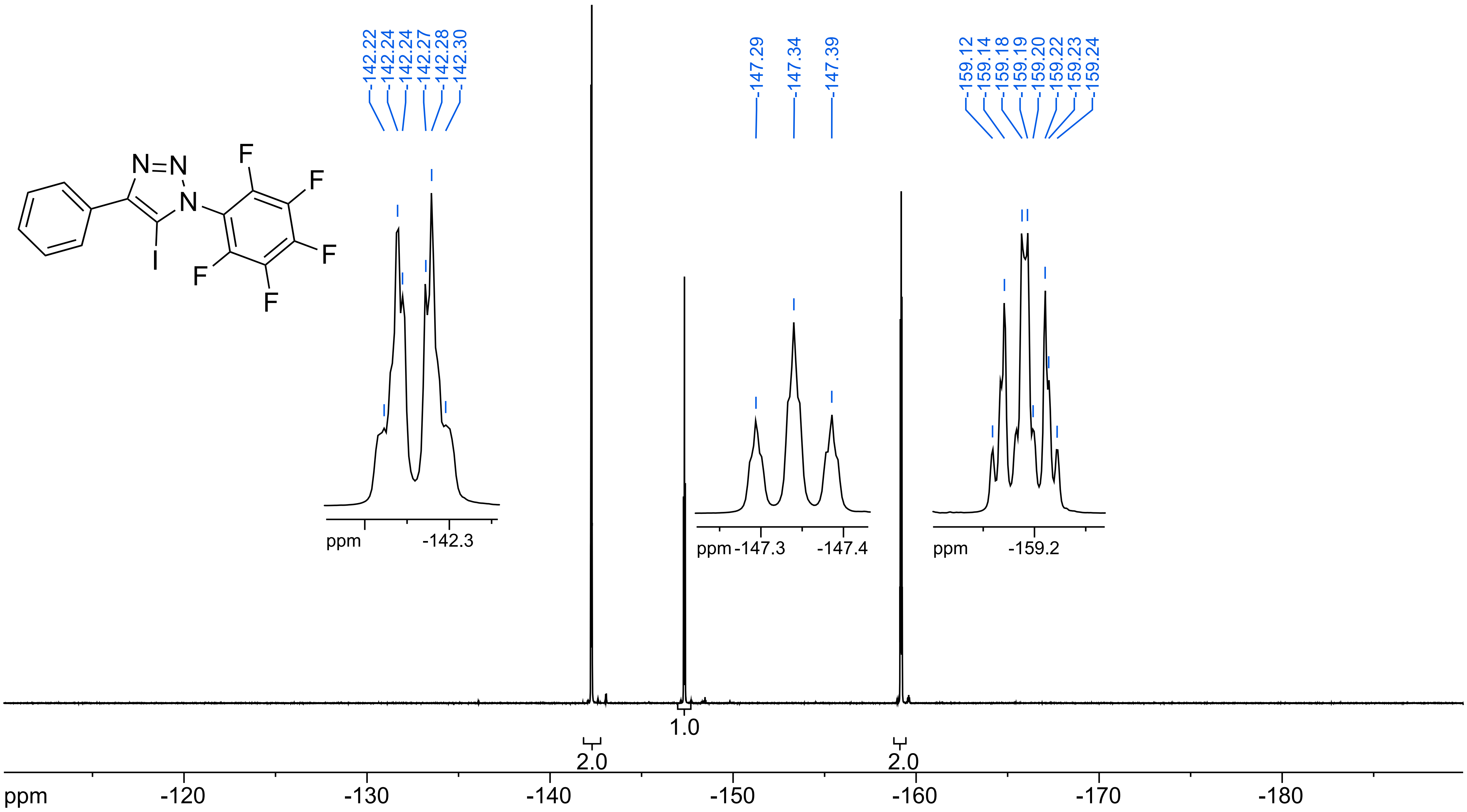

^{19}F NMR (470.5 MHz, RT, $CDCl_3$) spectrum of iodotriazole **1**

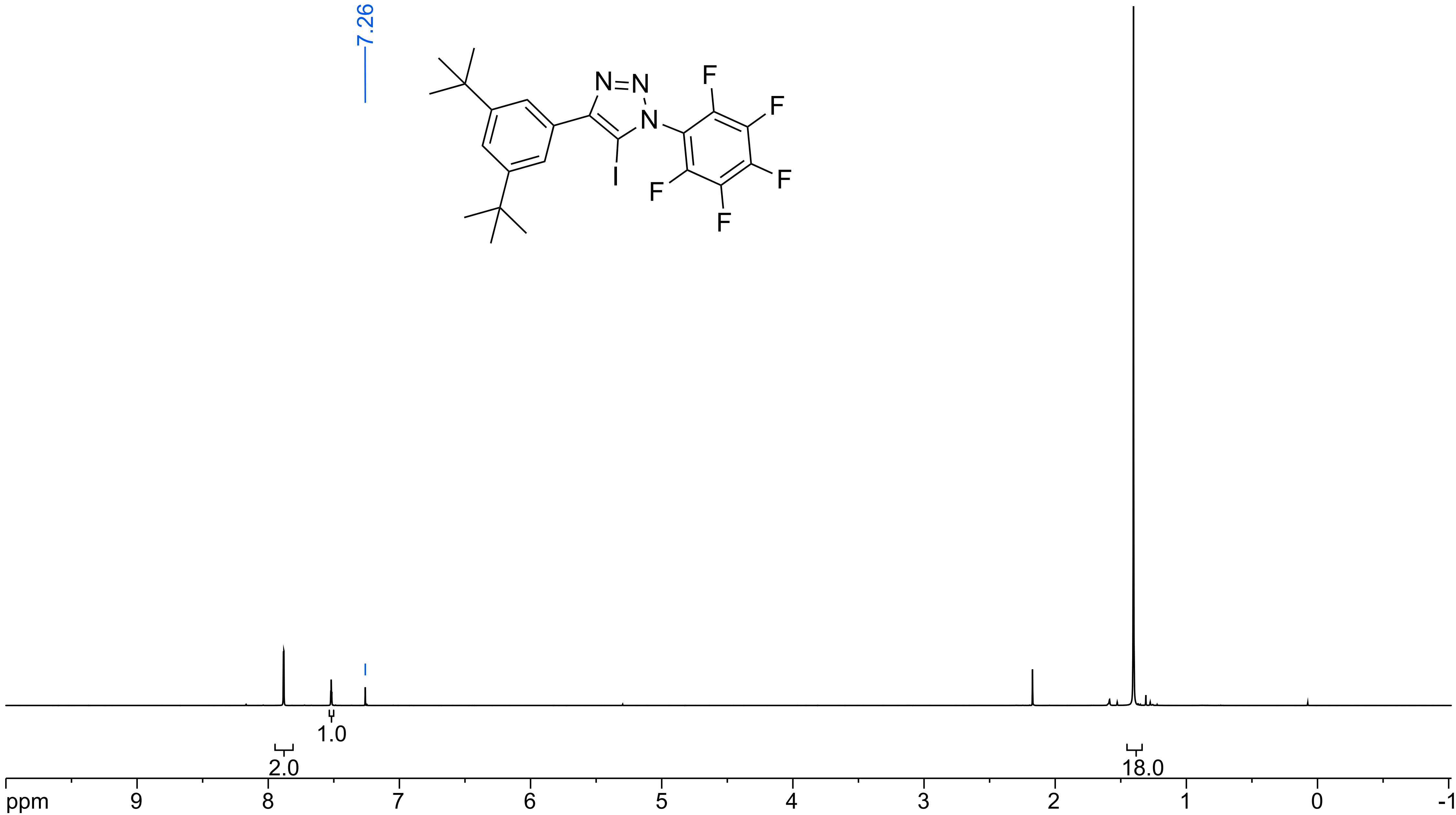

^{1}H NMR (500.1 MHz, RT, $CDCl_3$) spectrum of iodotriazole **7**

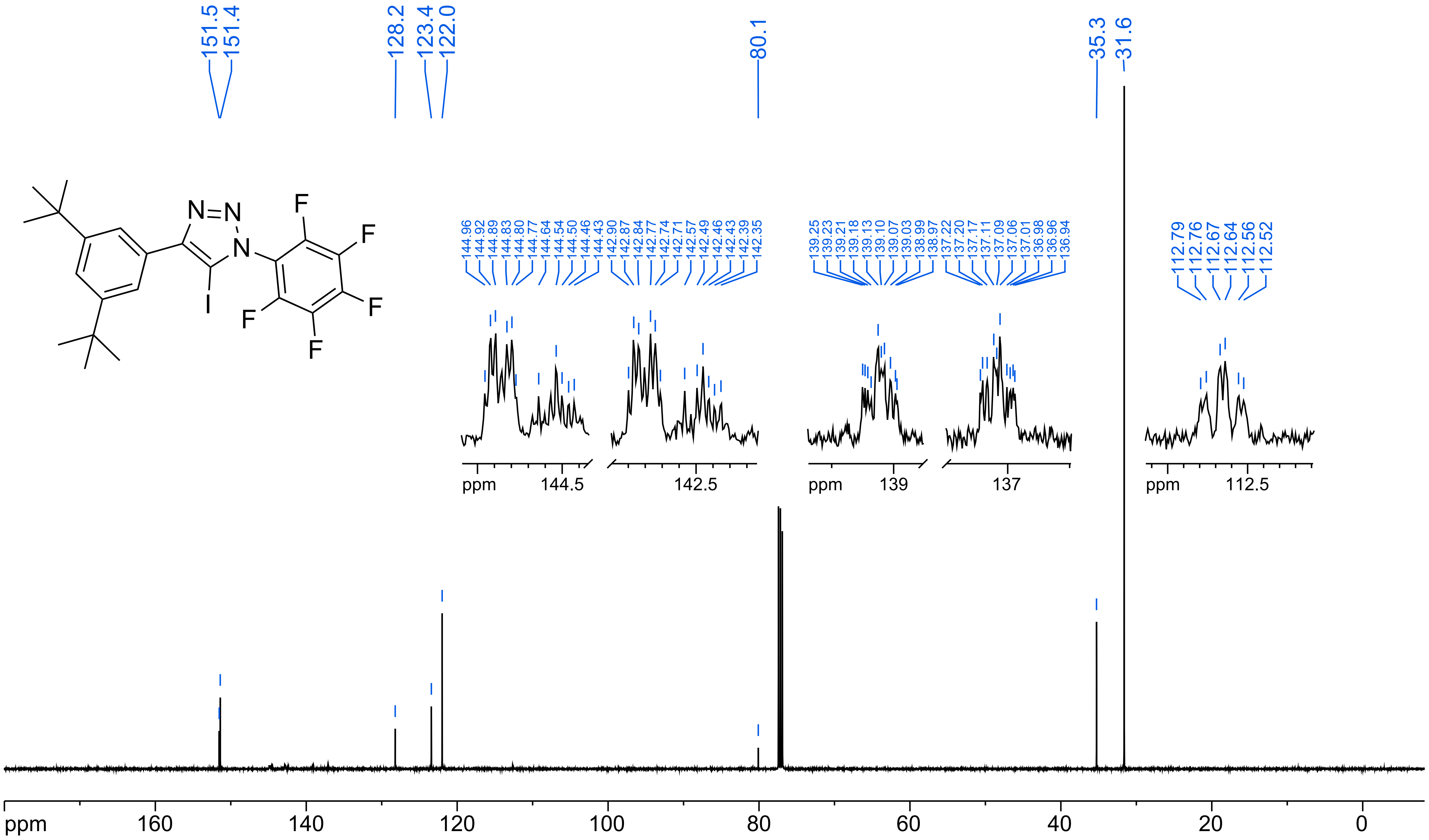

^{13}C NMR (125.7 MHz, RT, $CDCl_3$) spectrum of iodotriazole **7**

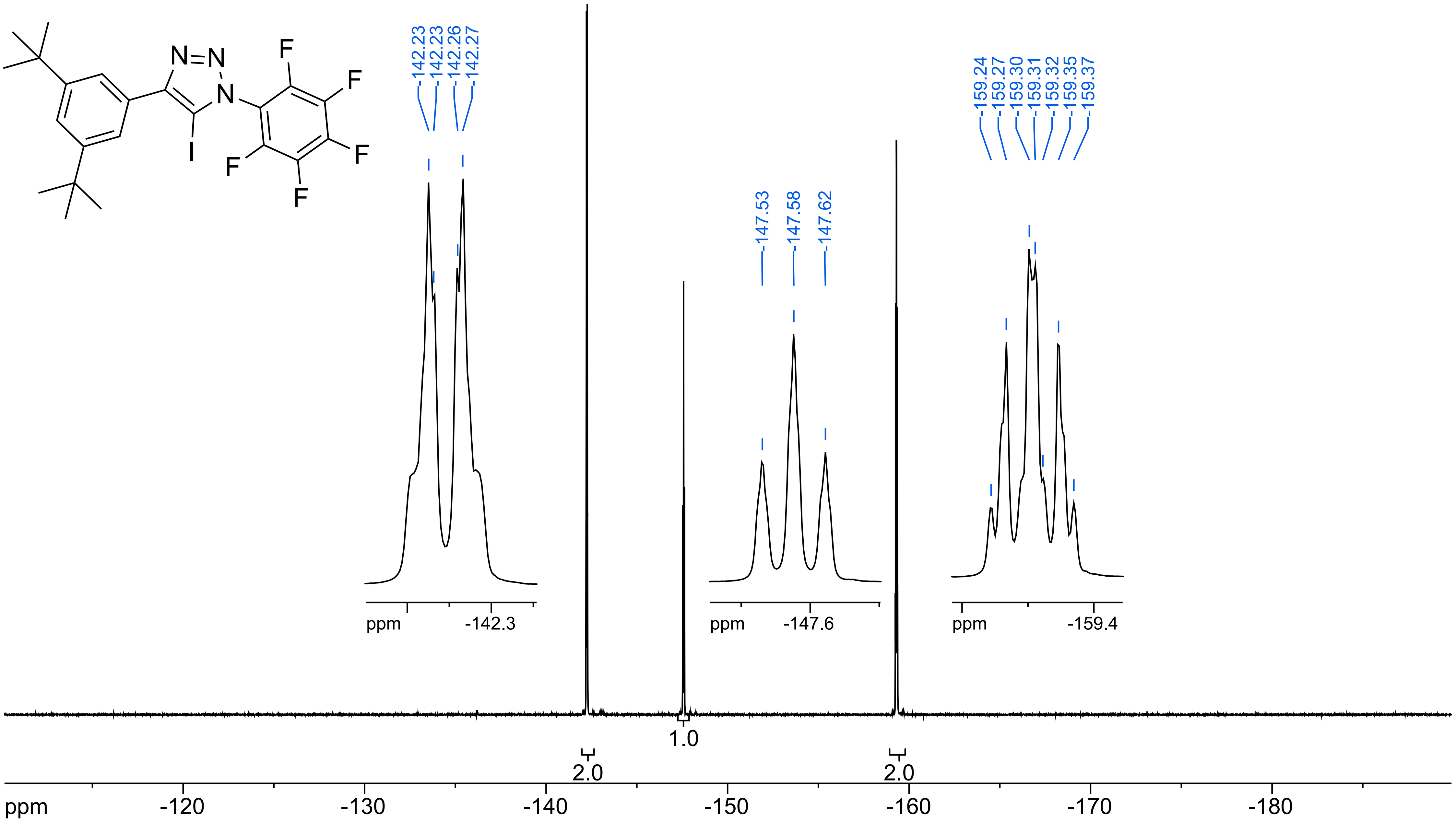

^{19}F NMR (470.5 MHz, RT, $CDCl_3$) spectrum of iodotriazole **7**

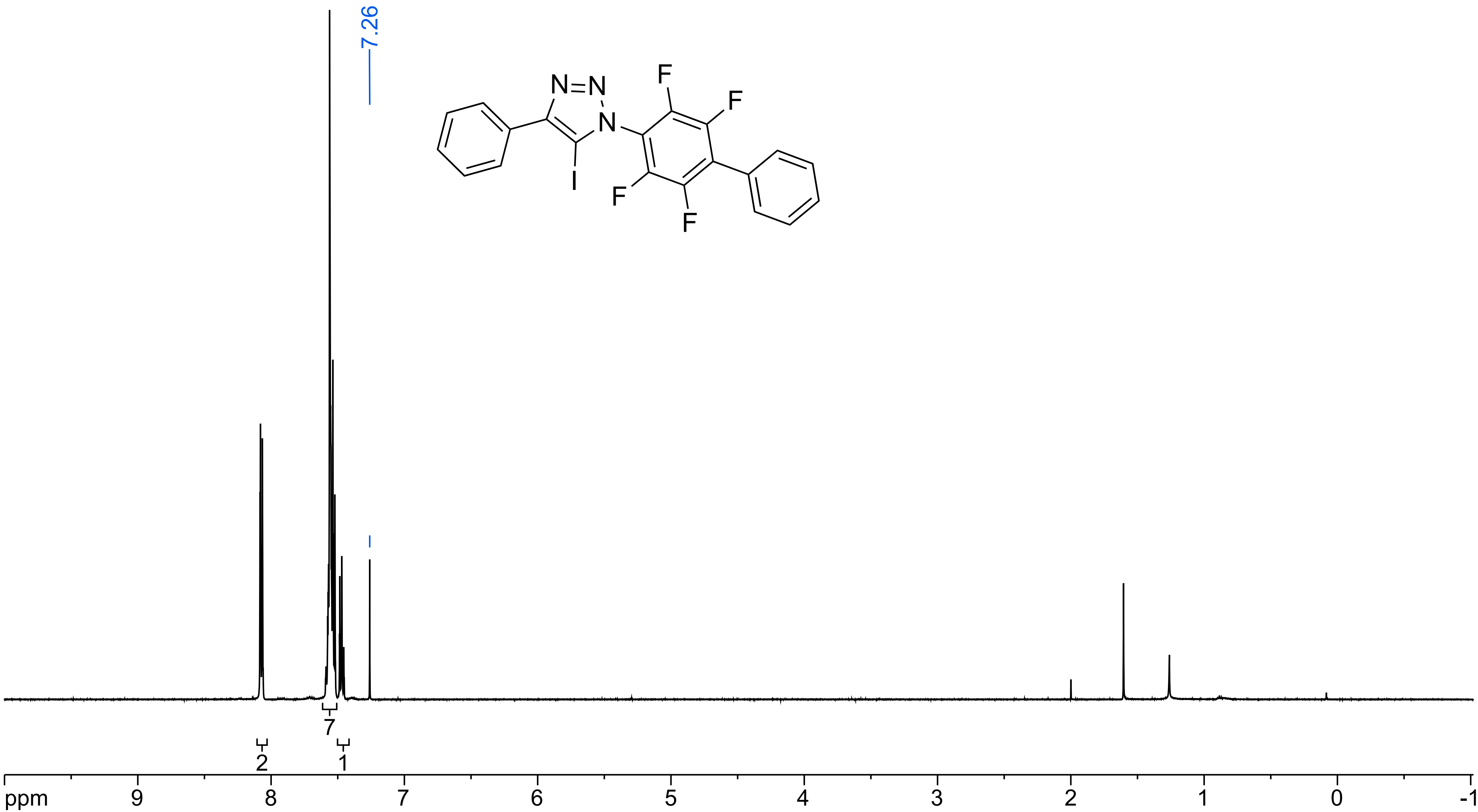

^{1}H NMR (500.1 MHz, RT, $CDCl_3$) spectrum of iodotriazole **S4**

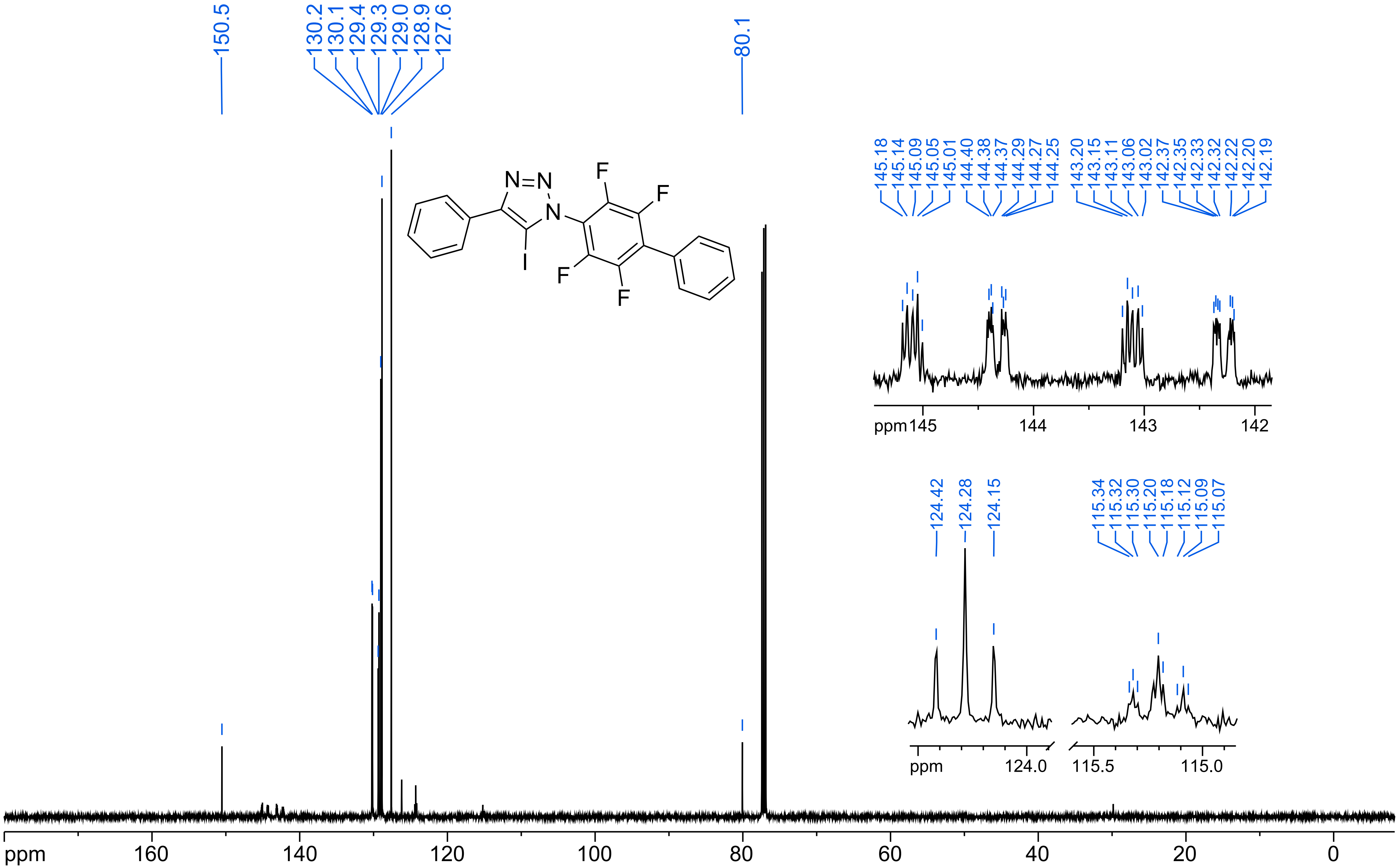

^{13}C NMR (125.7 MHz, RT, $CDCl_3$) spectrum of iodotriazole **7**

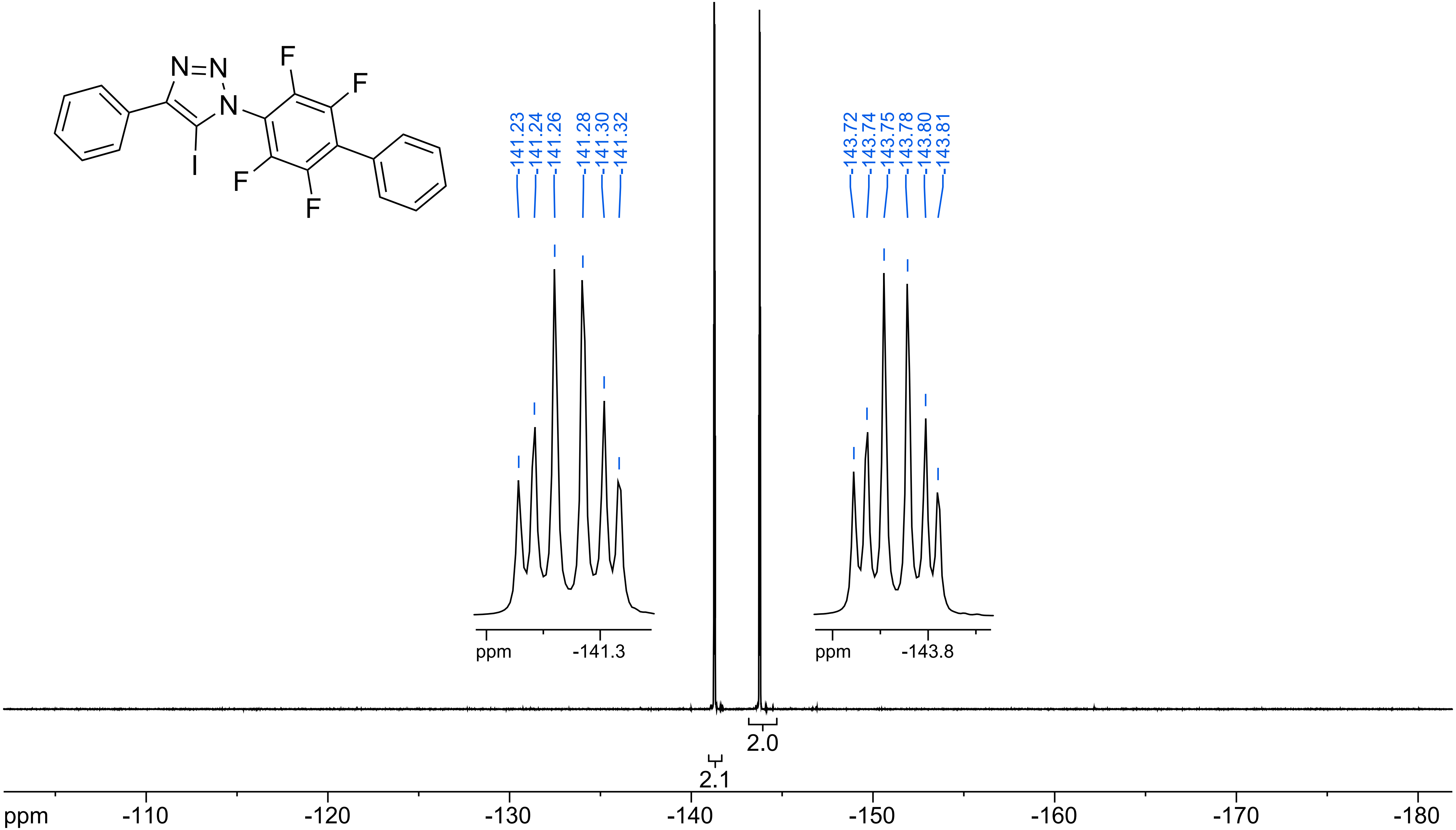

^{19}F NMR (470.5 MHz, RT, $CDCl_3$) spectrum of iodotriazole **S4**

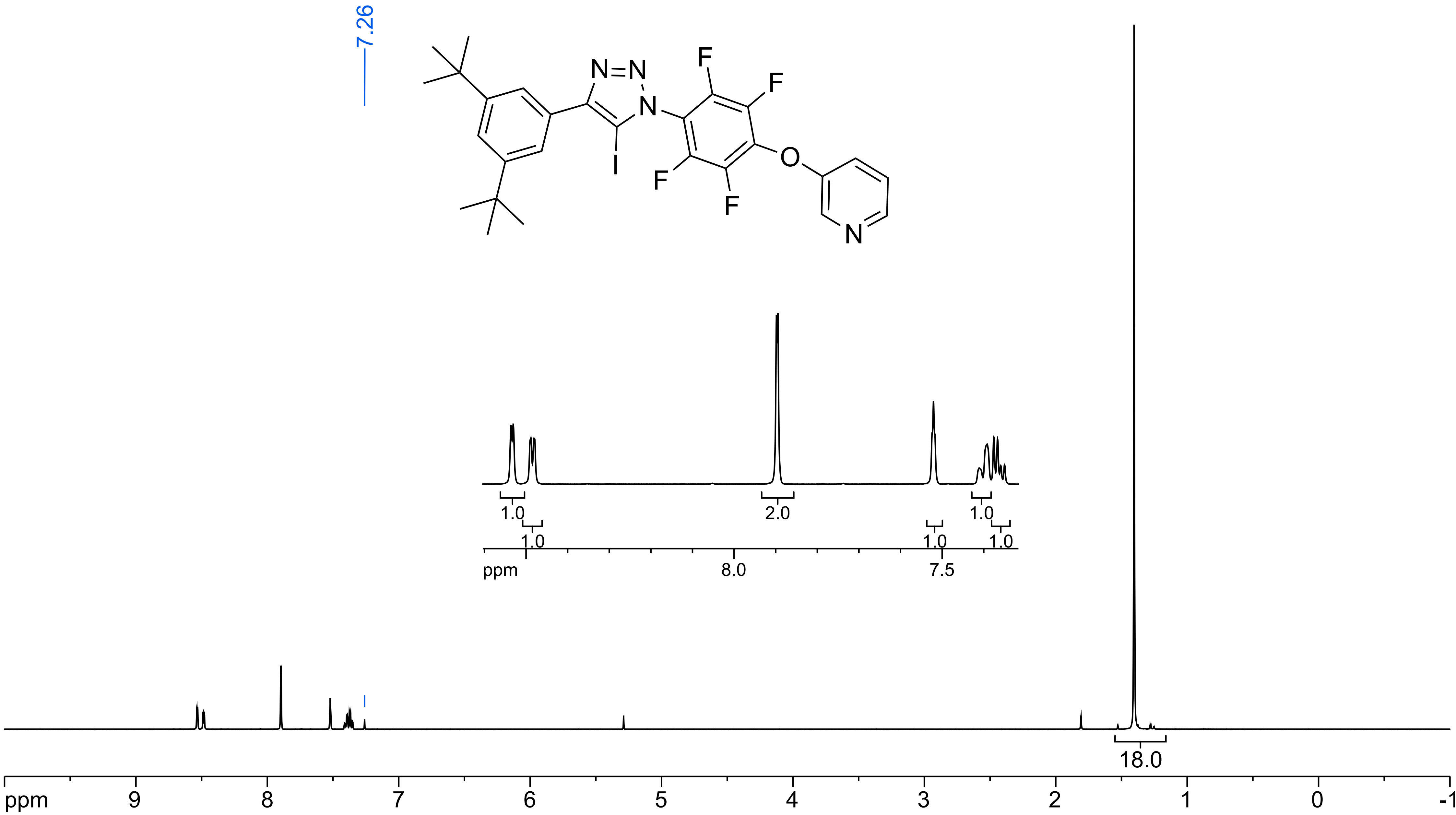

^{1}H NMR (500.1 MHz, RT, $CDCl_3$) spectrum of iodotriazole **9a**

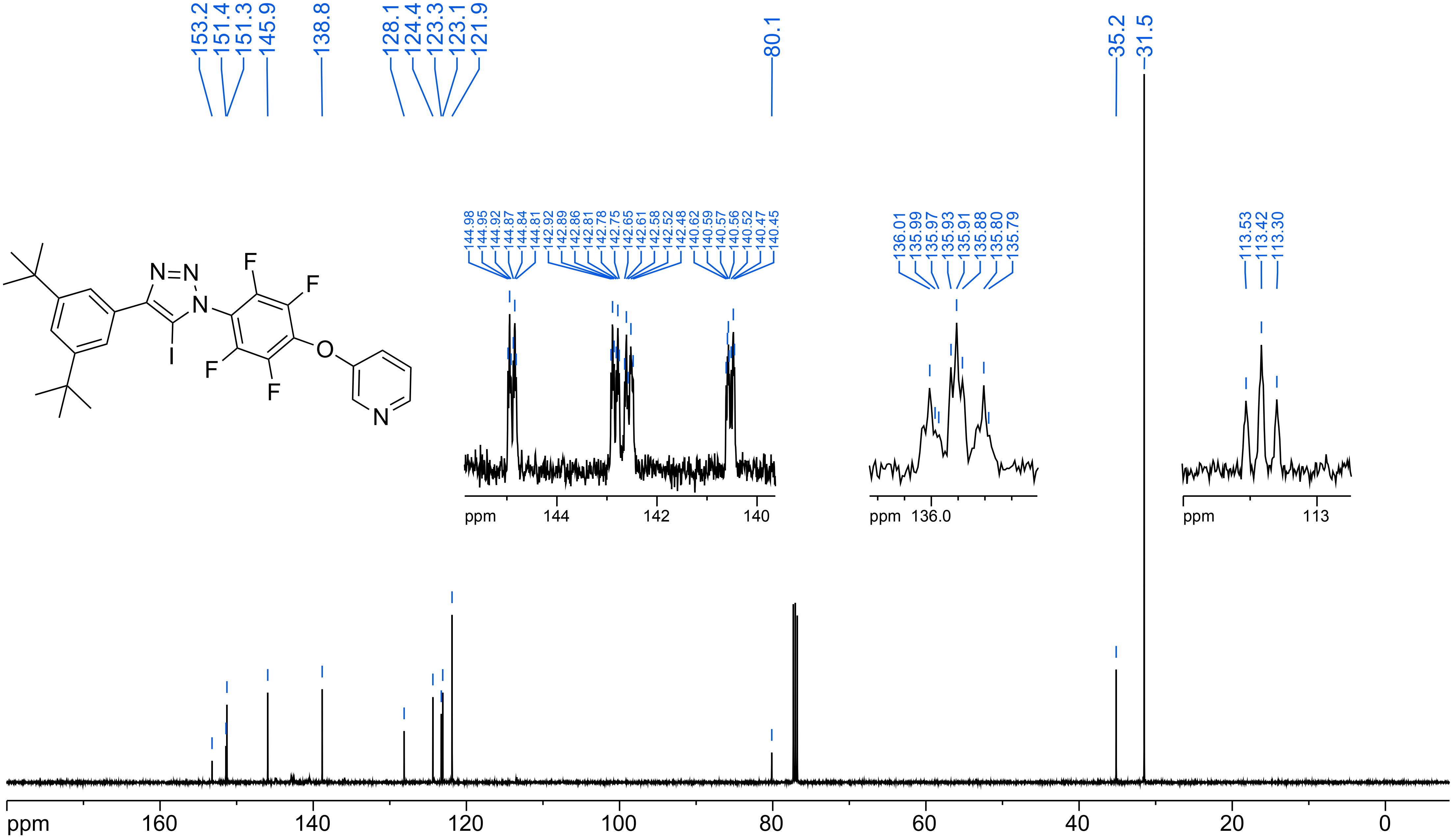

^{13}C NMR (125.7 MHz, RT, $CDCl_3$) spectrum of iodotriazole **9a**

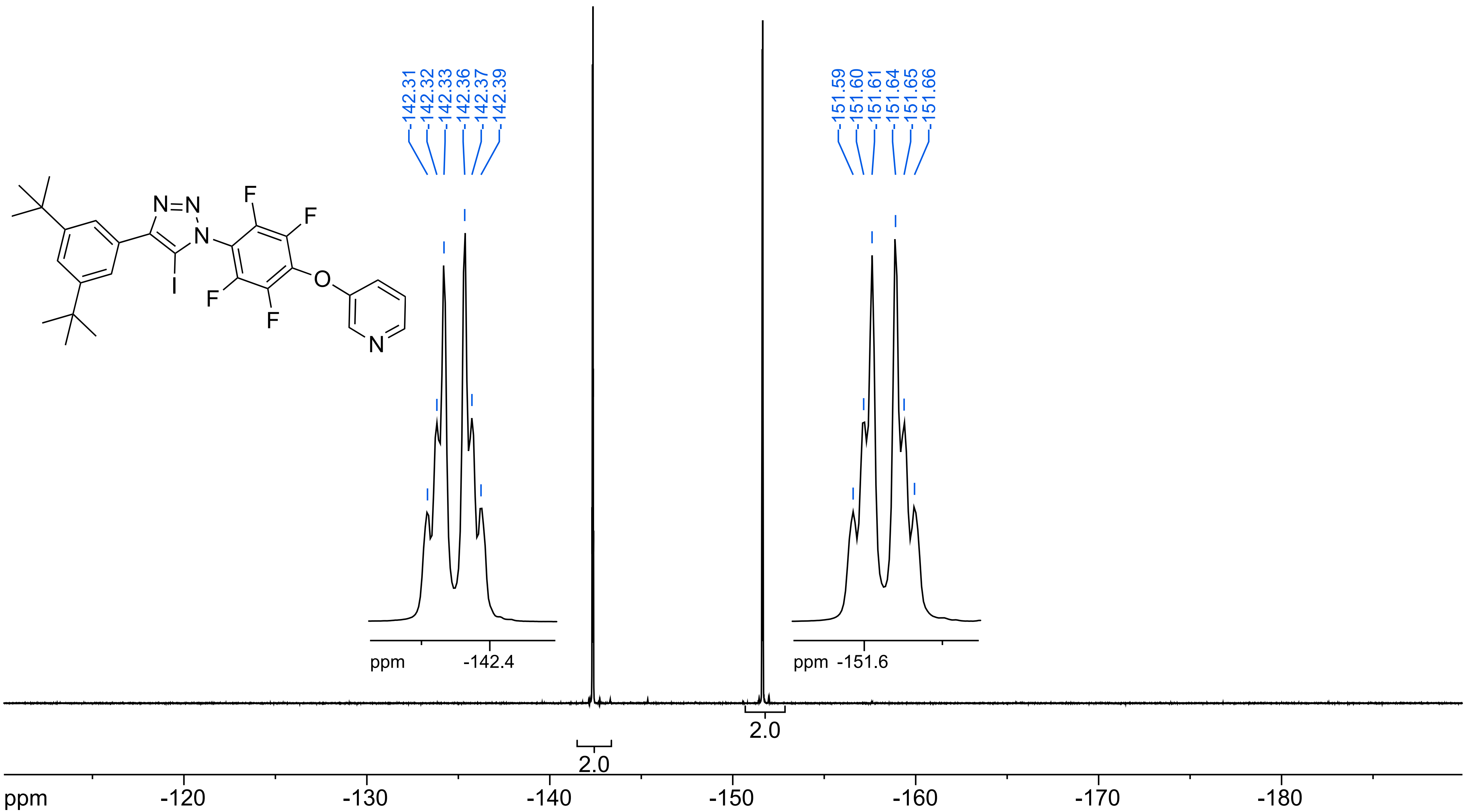

^{19}F NMR (470.5 MHz, RT, $CDCl_3$) spectrum of iodotriazole **9a**
